# Homeostatic Recovery of Embryonic Spinal Activity Initiated by Compensatory Changes in Resting Membrane Potential

**DOI:** 10.1523/ENEURO.0526-19.2020

**Published:** 2020-07-07

**Authors:** Carlos Gonzalez-Islas, Miguel Angel Garcia-Bereguiain, Peter Wenner

**Affiliations:** 1 Physiology Department, Emory University, School of Medicine, Atlanta, GA 30322; 2 Doctorado en Ciencias Biológicas, Univerisdad Autónoma de Tlaxcala, Tlaxcala 90070, Mexico; 3 One Health Research Group, Universidad de Las Americas, Quito 170505, Ecuador

**Keywords:** homeostatic, intrinsic plasticity, Na K ATPase, resting membrane potential, synpatic scaling, threshold voltage

## Abstract

When baseline activity in a neuronal network is modified by external challenges, a set of mechanisms is prompted to homeostatically restore activity levels. These homeostatic mechanisms are thought to be profoundly important in the maturation of the network. It has been shown that blockade of either excitatory GABAergic or glutamatergic transmission in the living chick embryo transiently blocks the movements generated by spontaneous network activity (SNA) in the spinal cord. However, the embryonic movements then begin to recover by 2 h and are completely restored by 12 h of persistent receptor blockade. It remains unclear what mechanisms mediate this early recovery (first hours) after neurotransmitter blockade, or even if the same mechanisms are triggered following GABAergic and glutamatergic antagonists. Here we find two distinct mechanisms that could underlie this homeostatic recovery. First, we see a highly robust compensatory mechanism observed shortly after neurotransmitter receptor blockade. In the first 2 h of GABAergic or glutamatergic blockade *in vitro*, there was a clear depolarization of resting membrane potential (RMP) in both motoneurons and interneurons. These changes reduced threshold current and were observed in the continued presence of the antagonist. Therefore, it appears that fast changes in RMP represent a key fast homeostatic mechanism for the maintenance of network activity. Second, we see a less consistent compensatory change in the absolute threshold voltage in the first several hours of *in vitro* and *in vivo* neurotransmitter blockade. These mechanisms likely contribute to the homeostatic recovery of embryonic movements following neurotransmitter blockade.

## Significance Statement

Homeostatic plasticity represents a set of mechanisms that act to recover cellular or network activity following a challenge and is thought to be critical for the developmental construction of the nervous system. The chick embryo afforded us the opportunity to observe the timing of homeostatic recovery of network activity following two distinct perturbations in a living developing system. Because of this advantage, we have identified a novel homeostatic mechanism that actually occurs as the network recovers and is therefore likely to contribute to nervous system homeostasis. We found that a depolarization of the resting membrane potential (RMP) and a hyperpolarization of threshold voltage in the first hours of the perturbation enhances excitability and supports the recovery of embryonic spinal network activity.

## Introduction

Recent work has focused on the mechanisms that allow networks to homeostatically maintain their activity levels in the face of various perturbations (Marder and Goaillard, 2006; Turrigiano, 2011; Davis, 2013). Typically, activity is altered for 24 h or more and compensatory changes in intrinsic cellular excitability and/or synaptic strength (synaptic scaling) are observed following the perturbation. While most of the work has been conducted *in vitro*, homeostatic mechanisms have also been observed *in vivo* in the spinal cord (Gonzalez-Islas and Wenner, 2006; Knogler et al., 2010), hippocampal (Echegoyen et al., 2007), auditory (Kuba et al., 2010), and visual systems (Desai et al., 2002; Goel et al., 2006). The chick embryo spinal cord expresses a spontaneously occurring network activity (SNA) that drives embryonic movements (O'Donovan, 1999; Blankenship and Feller, 2010). SNA likely occurs in all developing circuits shortly after synaptic connections form. In the embryonic spinal cord this activity is a consequence of the highly excitable nature of the nascent synaptic circuit where GABAergic neurotransmission is depolarizing and excitatory during early development (Ben-Ari et al., 1989; O'Donovan et al., 1998; O'Donovan, 1999; Rivera et al., 1999; Blankenship and Feller, 2010). Spinal SNA is known to be important in motoneuron axonal pathfinding (Hanson and Landmesser, 2004), and for proper muscle and joint development (Ruano-Gil et al., 1978; Toutant et al., 1979; Roufa and Martonosi, 1981; Persson, 1983; Hall and Herring, 1990; Jarvis et al., 1996).

The embryonic spinal cord provides an exceptional model of homeostasis. Many years ago, it was demonstrated that SNA expressed in the isolated spinal cord was transiently blocked by either glutamatergic or GABA_A_ receptor (GABA_A_R) antagonists, but within hours was homeostatically restored in the presence of that antagonist (Barry and O'Donovan, 1987; Chub and O'Donovan, 1998). However, the mechanisms of this recovery have not been identified. Interestingly, a similar homeostatic recovery of SNA-generated embryonic movements following neurotransmitter antagonists has also been demonstrated *in vivo* (Wilhelm and Wenner, 2008). When GABA_A_ or glutamate receptor antagonists were injected into the egg at embryonic day 8 (E8), SNA-driven embryonic movements were abolished for 1–2 h but then homeostatically recovered to control levels 12 h after the onset of pharmacological blockade of either transmitter (Wilhelm and Wenner, 2008). Therefore, it would be expected that mechanisms that contribute to the homeostasis of activity in the living system will have occurred by 2–12 h of treatment. Because the recovery was very similar following either GABAergic or glutamatergic blockade, one might think that similar mechanisms would drive the recovery of embryonic activity following injection of either antagonist, but this did not appear to be the case. It was determined that following 12 h of GABAR blockade compensatory changes in intrinsic excitability were observed (increased Na^+^ channel, and a decrease of two different K^+^ channel currents, I_A_ and Ik_Ca_), although changes in quantal amplitude were not observed until 48 h of receptor blockade (Wilhelm and Wenner, 2008; Wilhelm et al., 2009). On the other hand, following a 12-h glutamatergic blockade, no changes in intrinsic excitability were observed, and after 48 h of glutamatergic blockade, no change in quantal amplitude was seen.

Previous studies had not examined the possibility that compensatory changes in cell excitability and/or scaling were occurring at the onset and throughout the recovery process in motoneurons. In fact, very few studies have compared the expression of presumptive homeostatic mechanisms with the timing of the homeostatic recovery of activity, yet we would expect that some of these mechanisms would be expressed at the very onset of the recovery process. Further, there is little known about compensations that may be occurring in the interneurons that contribute to the drive of SNA. Therefore, we set out to identify the mechanisms that are expressed during the actual period of homeostatic recovery of SNA. We found some changes in threshold voltage, but importantly we describe a previously unrecognized mechanism of homeostatic intrinsic plasticity where fast changes in resting membrane potential (RMP) bring both interneurons and motoneurons closer to action potential threshold. The results suggest that compensatory changes in RMP could facilitate the homeostatic recovery of activity during glutamatergic or GABAergic blockade in the living embryo.

## Materials and Methods

### Dissection

E10 (or stage 36; Hamburger and Hamilton, 1951) chick spinal cords were dissected under cooled (15°C) Tyrode’s solution containing the following: 139 mm NaCl, 12 mm D-glucose, 17 mm NaHCO_3_, 3 mm KCl, 1 mm MgCl2, and 3 mm CaCl_2_; constantly bubbled with a mixture of 95% O_2_-5% CO_2_ to maintain oxygenation and pH around 7.3. After the dissection, the cord was allowed to recover overnight in Tyrode’s solution at 18°C. The next day, the cord was transferred to a recording chamber and continuously perfused with Tyrode’s solution heated to 27°C to allow for the expression of bouts of SNA with a consistent frequency.

### Electrophysiology

Whole-cell current clamp recordings were made from spinal motoneurons localized in lumbosacral segments 1–3 and were identified by their lateral position in the ventral cord. Recordings were also made from interneurons in the same segments, but these were identified by their more medial position in the ventral cord. Patch clamp tight seals (1–3 GΩ) were obtained using electrodes pulled from thin-walled borosilicate glass (World Precision Instruments, Inc) in two stages, using a P-87 Flaming/Brown micropipette puller (Sutter Instruments) to obtain resistances between 5 and 10 MΩ. Once whole-cell configuration was achieved, voltage clamp at –70 mV was maintained for a period of 5 min to allow stabilization before switching to current clamp configuration at which point the RMP was measured. A liquid junction potential of −12 mV was experimentally measured (Neher, 1992) for our conditions. All reported RMP and threshold values were then corrected offline. In some cases, whole-cell voltage clamp recordings were also obtained from motoneurons and interneurons to acquire miniature postsynaptic currents (mPSCs), and these recordings were obtained for the first 5–10 min of the recording before switching to current clamp to record measures of excitability. Series resistance during the recording varied from 15 to 20 MΩ among different neurons and was not compensated. Voltage clamp recordings were terminated whenever significant increases in series resistance (>20%) occurred or when holding current became larger than 50 pA. Cell capacitance was not compensated. Currents were filtered online at 5 kHz and digitized at 10 kHz. AMPA and GABA mPSCs were separated by their decay kinetics as described previously (Gonzalez-Islas and Wenner, 2006). The mPSCs with decay time constants (τ) under 7 ms were counted as AMPAergic, and those with τs over 10 ms were counted as GABAergic (Gonzalez-Islas and Wenner, 2006). We did not add tetrodotoxin (TTX) to isolate mPSCs in this study because the frequency and amplitude of spontaneous events (no TTX) and mPSCs in the presence of TTX have been shown to be the same (Chub and O'Donovan, 2001; Gonzalez-Islas and Wenner, 2006). The mPSCs were acquired on an Axopatch 200B patch clamp amplifier (Molecular Devices), digitized (Digidata 1200, Molecular Devices) on-line using PClamp 10 (Molecular Devices), and analyzed manually using Minianalysis software (Synaptosoft). For these recordings, if peak to peak noise was larger than 5 pA or the RMS was larger than 1 pA, then the recording was not included in the analysis. The mPSCs were identified automatically by Minianalysis using the following parameters: threshold, 5 pA; period to search a local maximum, 50 ms; time before a peak for baseline, 10 ms; period to search for a decay time, 35 ms; fraction of peak to find a decay time, 0.37 period to average a baseline 5 ms; area threshold 10; number of point to average peak 7; direction of peak; negative. We then went through these miniwaveforms and accepted them or rejected them following visual inspection of the waveform. Charts and associated average values were obtained by determining an average mPSC amplitude for each cell (variable number of mPSCs/cell, 5-pA cutoff), and then calculating the average of all cells. Recordings in current clamp were terminated whenever significant increases in input resistance (>20%) occurred. Current clamp recordings were filtered online at 10 kHz, digitized at 20 kHz. The intracellular patch solution for both current and voltage clamp recordings contained the following: 5 mm NaCl, 100 mm K-gluconate, 36 mm KCl, 10 mm HEPES, 1.1 mm EGTA, 1 mm MgCl_2_, 0.1 mm CaCl_2_, 1 mm Na_2_ATP, and 0.1 mm MgGTP; pipette solution osmolarity was between 280 and 300 mOsm, and pH was adjusted to 7.3 with KOH. Standard extracellular recording solution was Tyrode’s solution (see above), constantly bubbled with a mixture of 95% O_2_-5% CO_2_. In order to obtain rheobase, threshold voltage, and F-I relationships in embryos treated with saline or gabazine *in ovo*, a step protocol was employed (1-s duration, 1-pA increments for threshold/rheobase or 5-pA increments for the FI curve, at 0.1 Hz). To expedite this process so we could obtain more accurately timed measures of rheobase and threshold voltage following *in vitro* application of gabazine, a ramp protocol (from 0 to 200 pA; 1.2-s duration at 0.2 Hz; *n* = 3) was used. A test pulse was delivered 800 ms before every step pulse or ramp, a 200-ms hyperpolarizing current step of 20 pA was applied, and this provided our measure of input resistance and also served as an indicator of the reliability of the step. The RMP values were taken as an average of the V_m_ read at the beginning of each sweep in these protocols. Although most of the experiments were not blinded, in four experiments, the drug application *in ovo* was done blindly to corroborate the results.

### *In ovo* and *in vitro* drug injections

A window in the shell of the egg was opened to allow monitoring of chick embryo movements and drug application 6 or 12 h before isolating the spinal cord at E10. A total of 50 μl of a 10 mm gabazine solution was applied onto the chorioallantoic membrane of the chick embryo to a final concentration of ∼10 μm, assuming a 50-ml egg volume. For the *in vitro* drug application, 10 μm gabazine or 20 μm 6-cyano-7-nitroquinoxaline-2,3-dione disodium (CNQX) and 50 μm D-(-)−2-amino-5-phosphonopentanoic acid (APV) was added to the perfusate after recording from untreated/control neurons for the first 2–3 h.

### Recording of SNA

For monitoring SNA, tight-fitting glass suction electrodes were used to record ventrolateral funiculus (VLF) signals as described previously (O'Donovan and Landmesser, 1987). VLF signals were amplified (1000×), filtered (0.1 Hz to 1 kHz) by an extracellular amplifier (A-M Systems Inc.), and acquired using PClamp 10 (Molecular Devices). Analyses of the data were performed offline.

### Immunoblots

The ventral half of the lumbosacral spinal cords were homogenized in RIPA buffer containing protease and phosphatase inhibitors. Samples were then centrifuged to remove cell debris. Protein concentration was quantitated using BCA reagent (Pierce). Samples were separated on 4–15% SDS-PAGE and blotted to a nitrocellulose membrane. Films were scanned and analyzed using free software, ImageJ, with background correction and normalization to actin. The primary antibodies against Nav1.2 and Kv4.2 were from Alomone Labs. The blots were visualized by ECL chemiluminescence (GE Healthcare). Lysate from the ventral half of four different cords/chicks per treatment were used and blots were done in duplicate (total eight embryos per treatment).

### Drugs

SR-95531 hydrobromide (gabazine), CNQX, APV, and dihydro-β-erythroidine hydrobromide (DHβE) were purchased from Tocris Cookson (catalog numbers 1262, 1045, 0106, and 2349, respectively). All other chemicals and drugs were purchased from Sigma-Aldrich.

### Statistics

Data are expressed as mean ± SE. Statistical analysis of cellular excitability parameters was performed using ANOVA followed by Bonferroni *post hoc* test for multiple comparisons for normally distributed data and Kruskal–Wallis method followed by a *post hoc* Dunn test for data that was not normally distributed, unless mentioned otherwise. For statistical assessment of mPSC amplitude, we used a Student’s *t* test for normally distributed data, and Mann–Whitney test for data that was not normally distributed. For all of the experiments, the number of cells and cords are indicated in parenthesis at the bottom of the corresponding figure legend. Throughout the manuscript, *p ≤ 0.05, **p ≤ 0.01, and ***p ≤ 0.001.

## Results

### Changes in spinal neuron excitability in the first 12 h of *in vivo* GABAergic blockade

**Figure 1.**
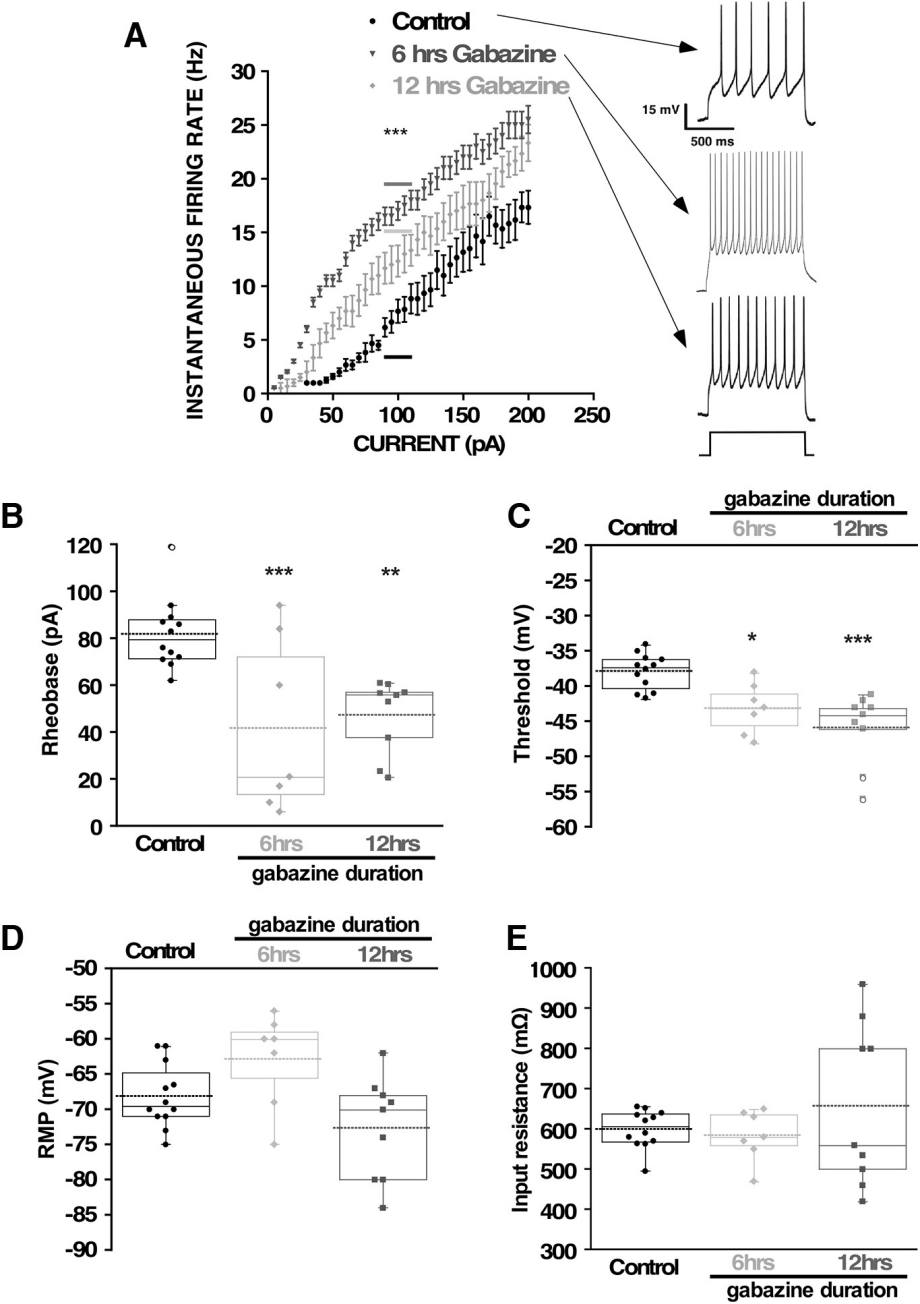
Changes in motoneuron excitability observed after chronic *in vivo* gabazine treatments. Motoneuron excitability was measured in isolated embryonic spinal cords by whole-cell current clamp using progressively more depolarized current steps to assess rheobase current and voltage threshold. Measurements were obtained from embryos that were untreated (12 cells, five cords), or treated for 6 h (seven cells, four cords) or 12 h (nine cells, five cords) with gabazine (10 μm at E9.5 or E9.75). ***A***, Average F-I curves for control motoneurons (*n* = 9), 6-h gabazine treatment (*n* = 6) or 12-h gabazine treatment (*n* = 9). Gabazine treatments shifted the average F-I curve to the left. All curves were significantly different from each other (values for steps of 90–110 pA were combined, horizontal bar, one-way ANOVA, Tukey’s *post hoc* test *p* < 0.001). The arrows point to representative traces for each condition evoked by current steps of 100 pA. Threshold current (***B***) or threshold voltage (***C***) was obtained by determining the minimum current necessary to evoke a spike in the recorded motoneuron. No significant changes in RMP (***D***) or input resistance (***E***) were found in the motoneurons recorded after chronic gabazine treatment at 6 or 12 h; *p < 0.05, **p < 0.01, ***p < 0.001.

Previous work showed that spinal motoneuron voltage-gated Na^+^ and K^+^ channel currents were altered following 12 h of *in ovo* GABAergic blockade, after embryonic movements had homeostatically recovered (Wilhelm et al., 2009). In order to determine whether these changes actually contribute to the recovery, we assessed cellular excitability in motoneurons during the period that the SNA-driven movements were actually recovering, but before complete recovery was achieved. First, we tested whether cellular excitability had increased during the period that movements were in the process of homeostatically recovering, following 6 h of gabazine treatment (10 μm) *in ovo*. We isolated the spinal cord following saline/gabazine treatment and recorded whole cell in current clamp from motoneurons that were no longer in the presence of gabazine. We found that threshold current (rheobase) was reduced and the absolute threshold voltage was hyperpolarized, suggesting the cells were more excitable following 6 h of gabazine treatment (Fig. 1*B*,*C*; Table 1). Following 12 h of gabazine treatment *in ovo*, similar changes were observed (Fig. 1*B*,*C*; Table 1). We also assessed excitability by giving current steps and plotting this against firing frequency after either 6 or 12 h of gabazine treatment (Fig. 1*A*). We saw a very strong shift toward higher excitability in the F-I curve at the 6-h time point, which then moved partly back toward pre-drug values following a 12-h treatment, although cells still showed a heightened excitability compared with controls. We did not observe changes in RMP or input resistance (Fig. 1*D*,*E*).

**Table 1:** Cellular excitability measures for motoneurons and interneurons following different treatments *in vitro* and in vivo

Condition	MotoneuronRheobase(pA)	MotoneuronV_m_ – V_t_(mV)	MotoneuronRMP (mV)	MotoneuronRm (MΩ)	InterneuronRheobase(pA)	InterneuronV_m_ – V_t_ (mV)	InterneuronRMP (mV)	Interneuron Rm (MΩ)
**Controls *in vivo***	81.8 ± 4.3	30.3 ± 1.6	–68.1 ± 1.3	599.6 ± 13.8	84.1 ± 8.3	34.7 ± 2.7	–70.2 ± 2.2	578.5 **±** 49.0
**GABA_A_R blockers6 h *in vivo***	**41.7 ± 14.0**	**19.7 ± 3.1**	–62.9 ± 2.5	584.3 ± 23.9	**29.1 ± 5.3**	21.5 ± 2.1	**–61.1 ± 1.7**	701.7 **±** 57.3
**GABA_A_R blockers12 h *in vivo***	**47.3±5.3**	26.8 ± 2.9	–72.7 ± 2.4	657.2 ± 67.3	**20.6 ± 3.7**	21.1 ± 2.3	–66.0 ± 2.2	624.8 ± 88.1
**Controls *in vitro***	74.3 ± 6.7	34.5 ± 2.1	–68.0 ± 1.4	537.2 ± 30.6	81.6 ± 5.7	33.7 ± 1.8	–71.3 ± 1.5	560.8 ± 40.9
**GABA_A_R blockers0–2 h *in vitro***	57.8 ± 7.1	20.3 ± 1.3	**–59.6 ± 1.8**	432.3 ± 37.3	**37.0 ± 6.9**	**16.3 ± 1.4**	**–55.6 ± 1.1**	494.6 ± 58.7
**GABA_A_R blockers2–4 h *in vitro***	**21.8 ± 7.2**	**28.1 ± 1.8**	**–58.6 ± 1.8**	474.4 ± 22.0	**35.7 ± 7.4**	**21.6 ± 1.4**	**–55.4 ± 1.3**	550.56 ± 70.0
**GABA_A_R blockers4–6 h *in vitro***	**32.1 ± 6.3**	**20.0 ± 1.4**	**–60.3 ± 1.1**	442.0 ± 41.0	**16.5 ± 2.5**	**15.8 ± 1.5**	**–60.3 ± 1.5**	582.5 ± 109.13
**GluR blockers0–2 h *in vitro***	46.6 ± 10.3	**18.6 ± 7.2**	**–57.4 ± 1.5**	492.7 ± 76.1	**34.3 ± 8.7**	**15.8 ± 1.1**	**–55.4 ± 1.1**	555.9 ± 56.4
**GluR blockers2–4 h *in vitro***	**27.6 ± 7.4**	**19.1 ± 1.8**	**–59.0 ± 1.8**	704.4 ± 54.3	**13.7 ± 2.7**	**14.6 ± 1.5**	**–53.7 ± 1.1**	687.1 ± 85.9
**GluR blockers4–6 h *in vitro***	**17.7 ± 4.2**	**16.1 ± 1.9**	**–58.0 ± 1.5**	**722.5 ± 54.1**	**12.2 ± 4.2**	**14.2 ± 1.1**	**–53.7 ± 0.9**	776.7 ± 72.9

Numbers in bold are show significant difference with controls. Measures are averages ± SEs.

**Figure 2.**
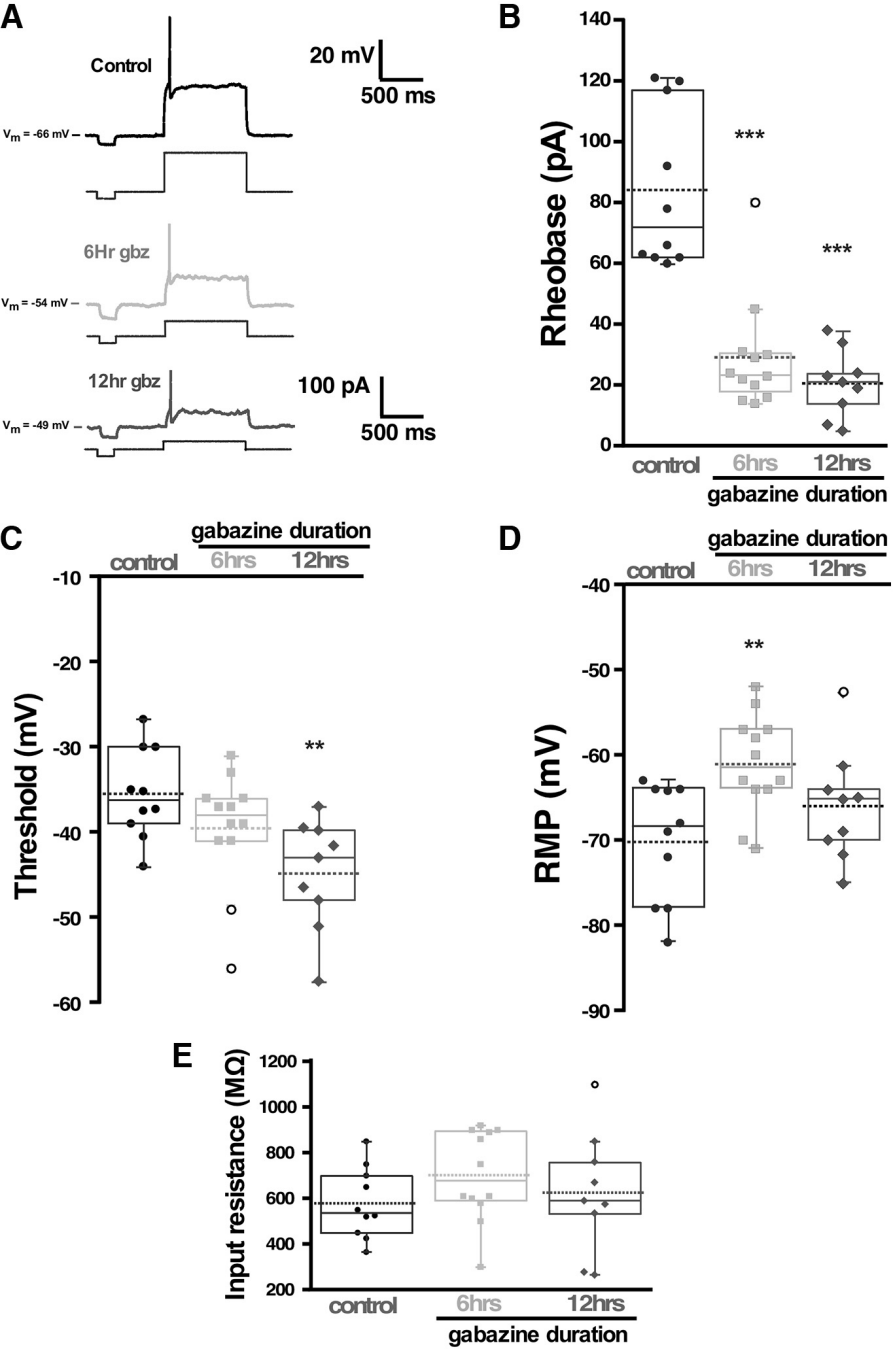
Interneuron excitability increased following *in vivo* GABAergic blockade. ***A***, Interneuron excitability was measured in isolated spinal cords from control or after *in ovo* chronic treatment with gabazine (10 μm) for 6 or 12 h. A step protocol (1 s, in 1-pA increments) was used to assess rheobase current and voltage threshold. Representative traces of interneuron firing in control (10 cells, five cords), or after 6 h (12 cells, four cords) and 12 h (*n* = 9, three cords) of chronic *in ovo* treatment with gabazine. Under each trace, the corresponding rheobase current applied to evoke firing is shown. RMP is also indicated at the left of each trace. Box and whisker plots superimposed to their corresponding dot plots show quartile distribution and individual values for Rheobase (***B***), spike threshold voltage (***C***), RMP (***D***), and input resistance (***E***). Kruskal–Wallis method followed by a *post hoc* Dunn test was used to assess statistical significance; **p < 0.01 and ***p < 0.001. For all plots, the continuous line represents the median and the dotted line represent the mean of the sample.

We wanted to determine whether these increases in cellular excitability were only occurring in motoneurons, or whether this was a more general phenomenon that extends to the rest of the developing motor circuitry. Thus, we assessed the possibility that spinal interneurons also increased cell excitability following gabazine treatment and could therefore contribute to the homeostatic recovery of SNA. Spinal neurons were targeted in the more medial positions of the cord. The population of spinal interneurons we recorded from were targeted blindly and therefore represent a diverse class of spinal interneurons with different neurotransmitters and activity patterns (Ritter et al., 1999). We found that, like motoneurons, interneurons had reduced threshold current at 6 and 12 h of gabazine treatment (Fig. 2*B*; Table 1). Threshold voltage was hyperpolarized at 12 h of gabazine treatment (Fig. 2*C*; Table 1). In addition, we did see a depolarization of the RMP at 6 h of treatment (Fig. 2*D*). Overall, the results suggest that there were increases in intrinsic excitability in motoneurons and interneurons at the point that embryonic movements were homeostatically recovering from *in ovo* GABAR blockade.

### Changes in spinal neuron excitability in the first 6 h of *in vitro* GABAergic blockade

**Figure 3.**
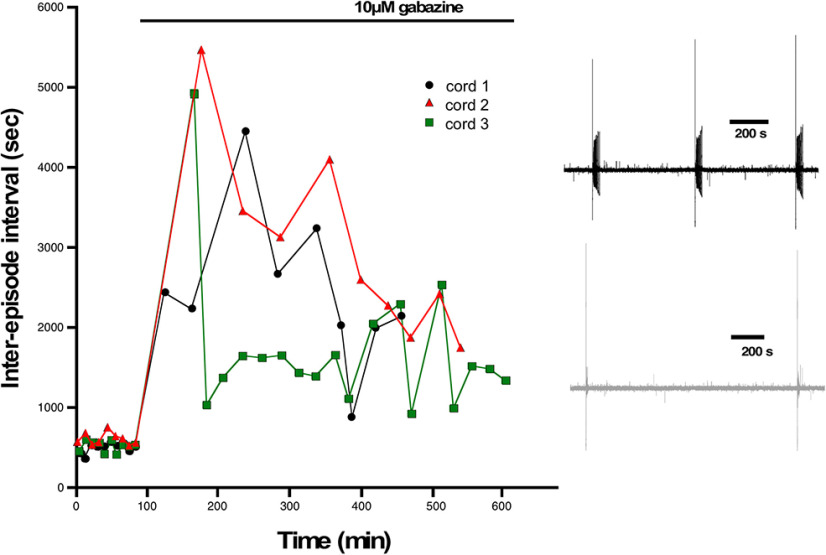
Episodes of SNA are abolished and then begin to recover following GABAergic blockade. The interval between episodes of SNA are plotted against elapsed time for three different cords before and following addition of gabazine to the bath. Episode intervals were increased following GABAergic block (10 μm gabazine), but then began to recover in the hours following gabazine. Inset shows example traces of SNA from a cord before (black) and after bath addition of gabazine (gray). Traces were filtered from 0.1 Hz to 10 kHz.

**Figure 4.**
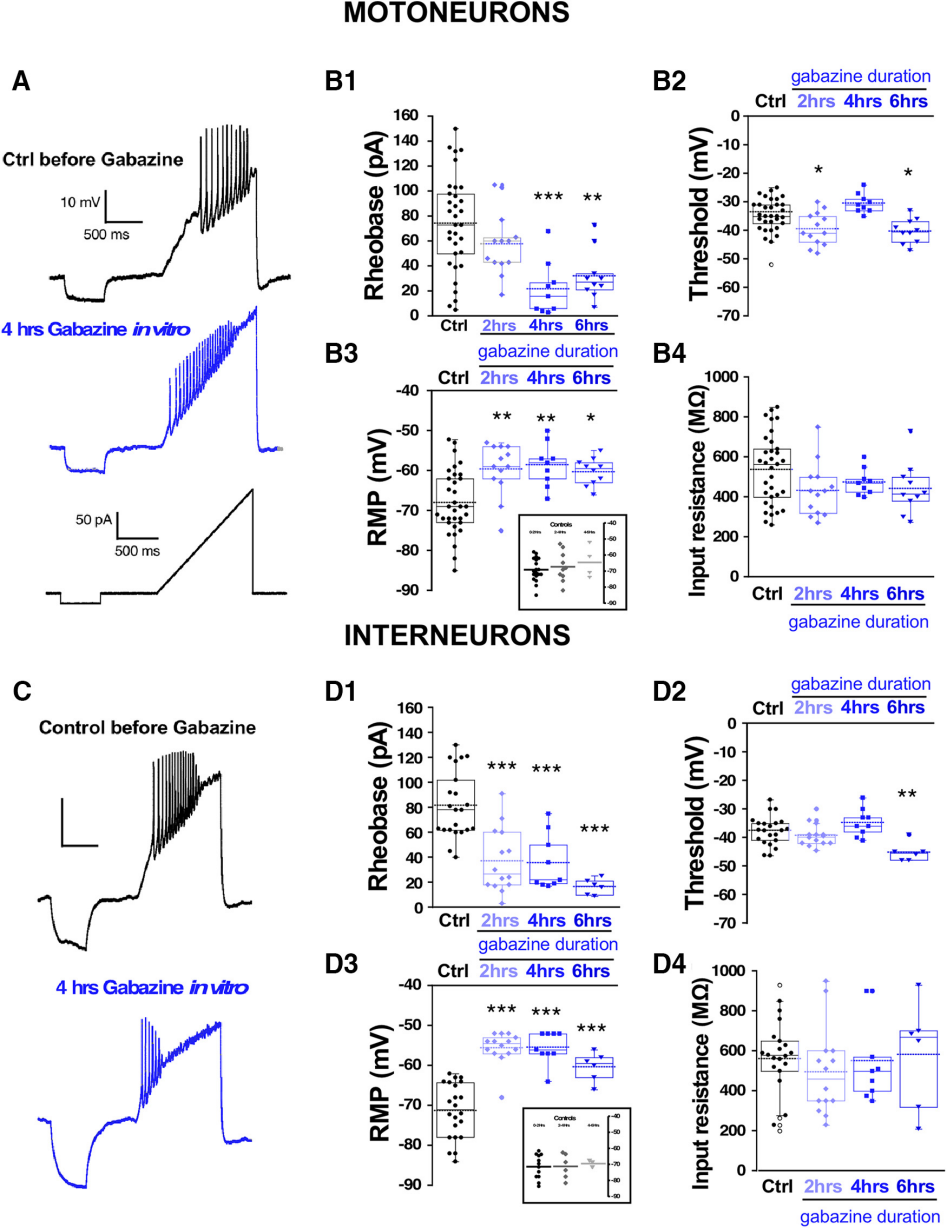
Spinal neuron excitability increased during the continuous blockade of GABARs *in vitro*. Motoneuron excitability was measured in isolated spinal cords using a ramp protocol from 0 to 200 pA in 1.2 s to assess rheobase current and voltage threshold in the absence (control, 33 cells; 12 cords) or in the continuous presence of gabazine 10 μM at three different blockade periods: 0–2 h (13 cells, four cords cells), 2–4 h (nine cells, three cords), and 4–6 h (10 cells, four cords). Representative traces of motoneuron firing in a control motoneuron or after 4 h in the continuous presence of gabazine in the bath. Lower trace shows the ramp protocol applied to evoke motoneuron firing (***A***). Box and dot plots showing quartile distribution and individual values for of the rheobase (***B1***), threshold (***B2***), RMP (***B3***), and input resistance (***B4***) in control; 0–2, 2–4, and 4–6 h in gabazine. Inset of ***B3*** shows results for cords that were never treated with gabazine, but cells were recorded in the first, second, or third 2-h periods. ***C***, ***D***, Interneuron excitability was measured in isolated spinal cords using the same protocol as above to assess rheobase and threshold in the absence (control, 22 cells; eight cords) or in the continuous presence of gabazine (10 μM) at three different blockade periods: 0–2 h (14 cells, five cords), 2–4 h (nine cells, three cords), and 4–6 h (six cells, three cords). Representative traces of firing in a control interneuron or after 4 h in the continuous presence of gabazine in the bath. Lower trace shows the ramp protocol applied to evoke interneuron firing (***C***). Box and dot plots showing the value of the rheobase (***D1***), threshold (***D2***), RMP (***D3***), or input resistance (***D4***) in control; 0- to 2-, 2- to 4-, and 4- to 6-h periods in the presence of gabazine. Inset of ***D3*** shows results for cords that were never treated with gabazine but interneurons were recorded in the first, second or third 2-h period. Kruskal–Wallis method followed by a *post hoc* Dunn test was used to asses statistical significance; *p < 0.05, **p < 0.01, ***p < 0.001. For all plots, the continuous line represents the median and the dotted line represent the mean of the sample.

One advantage of the earlier experiments was that the perturbation was conducted *in vivo*. Unfortunately, to measure cellular excitability, the cord must be isolated, and was given several hours to recover in the absence of gabazine before we could make excitability measurements. Such a process could itself alter the excitability of the cells. Therefore, in addition to the *in vivo* perturbations, we wanted to assess cellular excitability changes in the isolated cord *in vitro* in the first 6 h after adding the GABAR antagonist gabazine (10 μm). First, we added gabazine to the bath and observed its effect on the expression of SNA. Similar to previous work, we saw that episodes of SNA were initially blocked, but then began to recover in the following hours of GABAergic blockade (Fig. 3). As reported previously (Chub and O'Donovan, 1998), the duration of the episodes of SNA was reduced following bath addition of gabazine (Extended Data Fig. 3-1). To identify the mechanisms that recover SNA and which are expressed in the continued presence of gabazine we recorded whole cell in current clamp from motoneurons in the first (0–2), second (2–4), third (4–6) 2-h periods, and in cells that were never exposed to gabazine. Several aspects of cellular excitability were observed to increase in these first 6 h of GABAergic block. We saw a reduced threshold current (2–6 h; Fig. 4*B1*; Table 1), a hyperpolarized threshold voltage (0–2 and 4–6 h; Fig. 4*B2*; Table 1), and importantly, a fast ∼10 mV depolarization of the RMP (0–6 h; Fig. 4*B3*; Table 1). Cords that were never treated with gabazine *in vitro* but where motoneurons were recorded from 0 to 2, 2 to 4, or 4 to 6 h after warming the bath showed that the depolarized RMP was not simply a time-dependent process (Fig. 4*B3*, inset). Interestingly, we did not see a significant change in input resistance (Fig. 4*B4*; Table 1). These results suggest that compensatory changes in motoneuron excitability occurs very quickly and therefore could contribute to the recovery of SNA. The most striking compensatory change was a depolarizing shift in the RMP.

10.1523/ENEURO.0526-19.2020.f3-1 Extended Data Figure 3-1 SNA episode duration is reduced following neurotransmitter receptor blockade. Following the bath addition of gabazine, episode duration is reduced compared to that before adding the drug. Individual dots represent duration of a single episode before and after drug addition to an individual cord. Download Figure 3-1, TIF file.

Similar increases in cellular excitability were observed in interneurons from isolated cords that were treated with bath application of gabazine for 0–2, 2–4, and 4–6 h *in vitro* (Fig. 4*D1–D4*; Table 1). Cords that were never treated with gabazine but where interneurons were recorded from 0 to 2, 2 to 4, or 4 to 6 h after warming the bath showed that the depolarized RMP was not simply a time-dependent process (Fig. 4*D3*, inset). Because we were recording from diverse classes of spinal neurons, the results suggest that various cell types alter their cellular intrinsic excitability and contribute to the recovery of activity following GABAergic blockade. Importantly, interneuron RMP was significantly depolarized at each of the time points (Fig. 4*D3*). No changes were observed in input resistance in any condition. Therefore, interneurons increased their cellular excitability after GABAergic blockade similarly to motoneurons.

**Figure 5.**
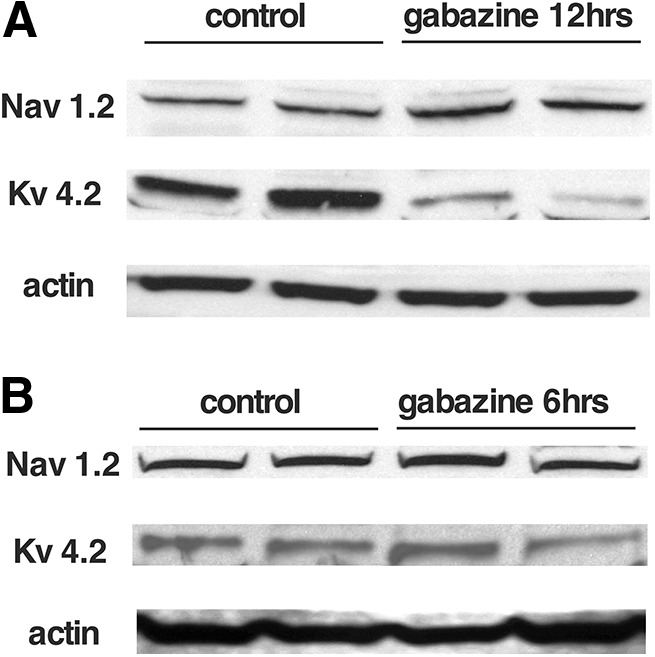
Changes in voltage-gated channel expression following *in ovo* gabazine treatment. Western blottings showing changes in the expression of voltage-dependent Na^+^ channels (Nav1.2) and inactivating K^+^ channels (Kv4.2) in the chick embryo spinal cord following 12 h (***A***) or 6 h (***B***) of GABAR blockade *in ovo*.

Since the changes in cellular excitability following GABA_A_R blockade appear to be expressed across multiple cell types throughout much of the cord, we ran Western blottings of isolated spinal cords (ventral half) and assessed 2 of the voltage-gated channels that we expected could mediate this process. It has been reported (Wilhelm et al., 2009) that gabazine-induced changes were observed in voltage-gated Na^+^ and K^+^ channels. In that study, we saw TTX-sensitive voltage-gated Na^+^ channel currents were increased. Therefore, we assessed the levels of Nav1.2, an α subunit of the voltage-gated Na^+^ channel, which had been shown to be expressed early in the development of the embryonic chick (Kuba et al., 2014). We found that following a 12-h gabazine treatment *in ovo*, Nav1.2 expression was increased (172.4 ± 14.8%, *p* ≤ 0.05) but not after a 6-h treatment (105.3 ± 5.3%; Fig. 5). Further, it was also observed in that study that currents of the A-type transiently-activated K^+^ channel (I_A_) and the calcium-dependent K^+^ channel (Ik_Ca_) were both decreased following gabazine treatment (Wilhelm et al., 2009). Here, we show that expression of Kv4.2 (which mediates the A-type K^+^ channel in chick embryo; Dryer et al., 1998) is downregulated following a 12-h gabazine treatment (54.5 ± 2.5%, *p* ≤ 0.05) but not after 6 h (104.2 ± 17.2%; Fig. 5). Together, the results show that cellular excitability is altered during and after the homeostatic recovery of SNA and that expression changes in two different voltage-gated channels do not occur until later stages of the recovery.

### The trigger for changes in RMP was distinct from the homeostatic mechanisms expressed after GABAergic blockade

**Figure 6.**
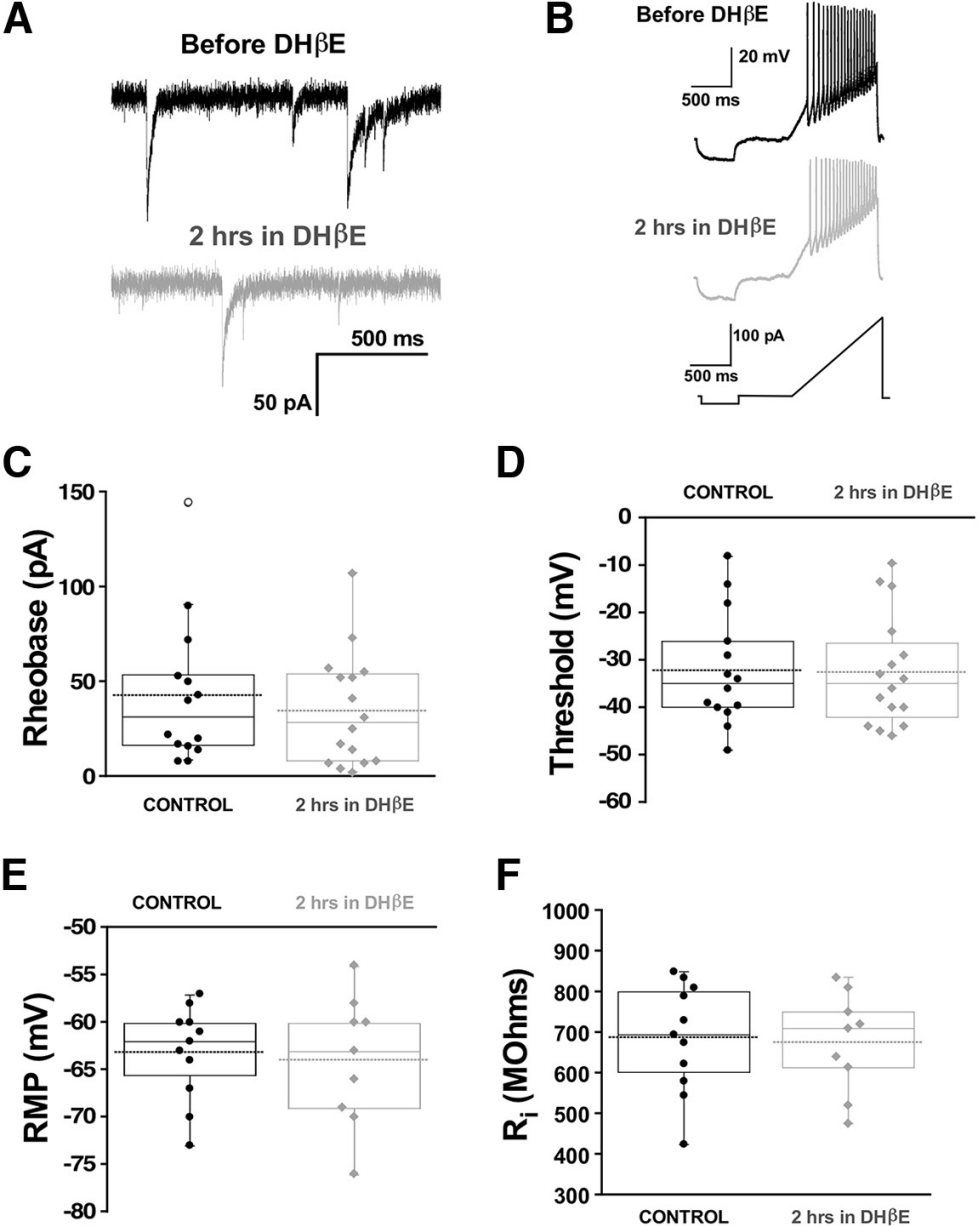
Chronic reductions in quantal GABA release that mediate synaptic upscaling do not trigger changes in motoneuron excitability. Whole-cell voltage and current clamp recordings from motoneurons were obtained from isolated spinal cords before (14 cells, six cords) or after 2 h of the nicotinic receptor antagonist (16 cells, six cords) DHβE (10 μM). Representative traces are shown. Top black trace, mPSC before DHβE addition; bottom gray trace, following nicotinic receptor inhibition with DHβE addition (***A***). Despite this, no changes in intrinsic cell excitability were measured after 2 h of DHβE treatment. Whole-cell current clamp recording during a ramp test showing representative traces of motoneurons, before and after 2 h of DHβE (***B***). The ramp protocol was applied to evaluate rheobase current and voltage threshold. Box and dot plots show that rheobase (***C***) threshold (***D***), RMP (***E***), and input resistance (***F***) were not different after 2 h of DHβE compared with controls. A Mann–Whitney test was used to quantified statistical significance. For all plots, the continuous line represents the median and the dotted line represent the mean of the sample.

Following GABA_A_R blockade, compensatory changes in synaptic strength (scaling) were not observed until 48 h, but voltage-gated conductance changes were triggered by 12 h (Wilhelm and Wenner, 2008; Wilhelm et al., 2009). It has been recently reported that simply reducing GABA_A_R activation due to spontaneous miniature release of GABA vesicles (spontaneous GABAergic transmission) was sufficient to trigger upscaling (Garcia-Bereguiain et al., 2016). We were able to do this by taking advantage of our observation that manipulating nicotinic receptor activation altered spontaneous GABAergic release (Gonzalez-Islas et al., 2016). In this previous study, we showed that the nicotinic antagonist DHβE reduces GABA_A_, but not AMPA, mPSCs by ∼30%. Therefore, we tested the possibility that the fast changes in cellular excitability observed in the current study were also mediated by reduced spontaneous miniature GABAergic neurotransmission. Whole-cell recordings from motoneurons were obtained before and 2 h after DHβE application *in vitro*. We did not find any differences in cellular excitability following reduction of GABA quantal release by DHβE (Fig. 6). Therefore, unlike the trigger for synaptic scaling, the compensatory changes in cellular excitability in the first hours of GABAergic blockade were not mediated by changes in spontaneous GABAergic transmission.

### Synaptic scaling does not contribute to the homeostatic recovery of SNA-generated movements

**Figure 7.**
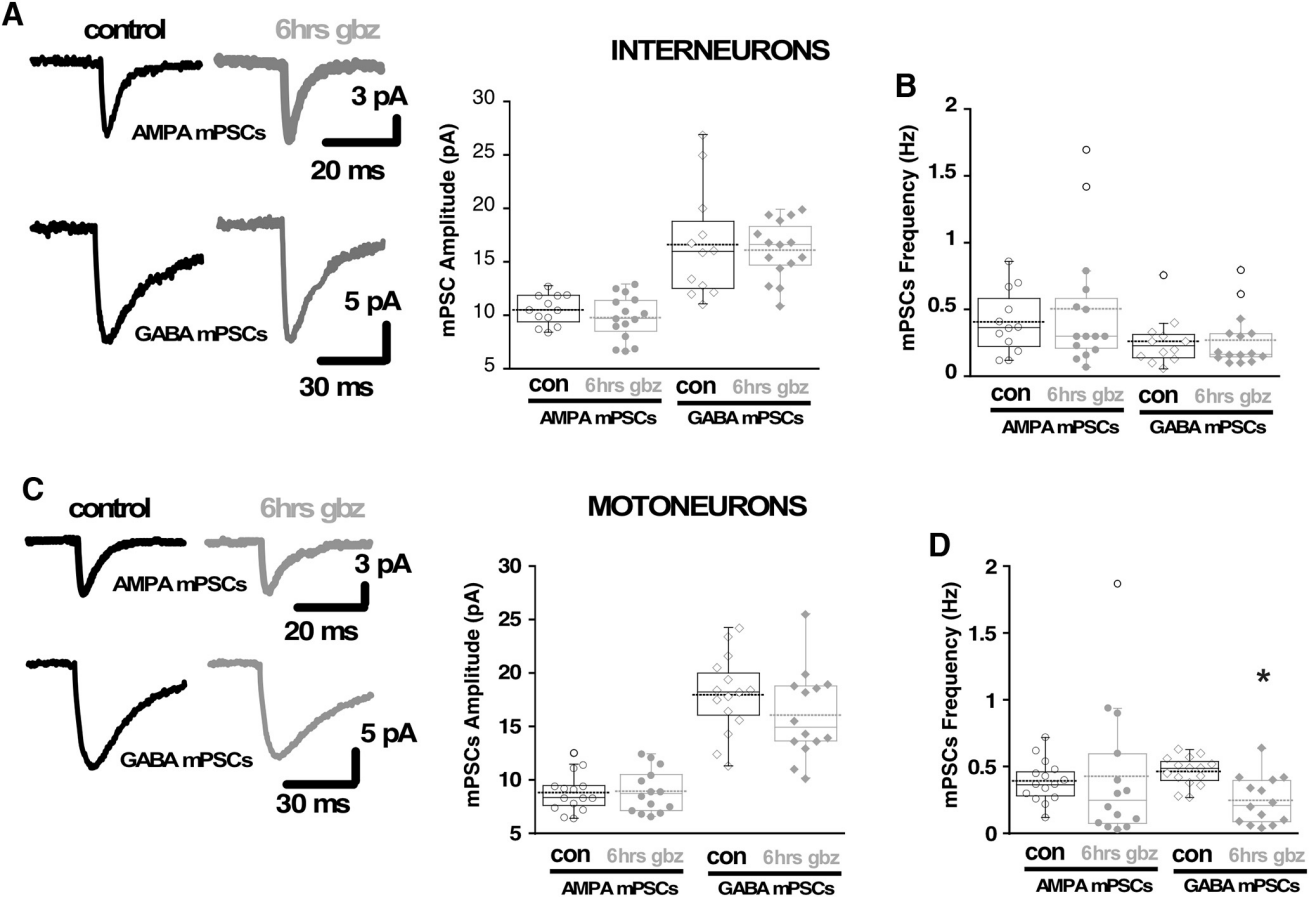
Synaptic scaling does not contribute to the homeostatic recovery of SNA-generated movements. ***A***, Representative traces of the average mPSCs from single interneurons in control conditions or after 6 h of *in vivo* gabazine treatment (left side). Box and dot plots (right side) showing quartile distribution and individual values for AMPAergic and GABAergic mPSC amplitudes from embryonic spinal interneurons in control conditions (12 cells, four cords) and after 6 h of chronic treatment with 10 μm gabazine (15 cells, five cords). ***B***, Box and dot plots showing AMPAergic and GABAergic mPSC frequency from embryonic spinal interneurons neurons in control conditions and after 6 h of chronic treatment with gabazine. ***C***, Representative traces of the average mPSCs from single motoneurons in control conditions or after 6 h of gabazine treatment (left side). Box and dot plots (right side) showing quartile distribution and individual values for AMPAergic and GABAergic mPSC amplitudes from embryonic spinal motoneurons in control conditions (15 cells, five cords) and after 6 h of chronic treatment with 10 μm gabazine (14 cells, five cords). ***D***, Box and dot plots showing AMPAergic and GABAergic mPSC frequency from embryonic spinal motoneurons in control conditions and after 6 h of chronic treatment with gabazine. For all plots, the continuous line represents the median and the dotted line represent the mean of the sample. *p < 0.05.

Previously, it had been shown that AMPAergic and GABAergic upscaling were not observed in chick embryo motoneurons following a 12-h gabazine treatment *in ovo* (Wilhelm and Wenner, 2008). It remained possible that interneurons experienced scaling and contributed to the homeostatic recovery of SNA in the first hours of gabazine treatment. However, following 6 h of gabazine treatment *in ovo*, we found no change in interneurons in AMPA mPSC amplitude (Fig. 7*A*) or decay kinetics (Extended Data Fig. 7-1). We would not have expected GABAergic scaling to contribute to the recovery of movements as we were blocking GABARs. Regardless, we did not see any change in GABA mPSC amplitude (Fig. 7*A*) or decay kinetics (Extended Data Fig. 7-1) in interneurons following a 6-h gabazine treatment *in ovo*. The results show that neither AMPAergic nor GABAergic scaling in interneurons contributed to the homeostatic recovery of activity levels.

10.1523/ENEURO.0526-19.2020.f7-1 Extended Data Figure 7-1 Decay time constants (τ) for GABA mPSCs (***A***) before (τ =17.06 ± 0.83 ms; *n* = 15) and (***B***) after (τ = 15.7 ± 0.86 ms; *n* = 14) the addition of gabazine to the bath were not significantly different (*p* = 0.26). τ for AMPA mPSCs (***C***) before (τ = 3.16 ± 0.19 ms; *n* = 15) and (***D***) after addition of gabazine to the bath (τ = 3.39 ± 0.17 ms; *n* = 14) are shown. No significant difference was observed (*p* = 0.39). Download Figure 7-1, TIF file.

It was shown previously that scaling is not triggered in motoneurons after 12 h of gabazine treatment (Wilhelm and Wenner, 2008). We tested whether AMPAergic and GABAergic scaling was expressed in motoneurons following 6 h of gabazine treatment *in ovo*. We found no difference in AMPAergic or GABAergic mPSC amplitude (Fig. 7*C*) or decay kinetics (Extended Data Fig. 7-1) from controls. These findings showed that synaptic scaling of AMPAergic mPSCs in different spinal populations could not have contributed to the recovery of SNA or the movements it drives following GABAergic blockade. Finally, there was no compensatory increase in mPSC frequency in interneurons (Fig. 7*B*), or motoneurons (Fig. 7*D*). In fact, we found a significant reduction in GABAergic mPSC frequency.

### Recovery of embryonic movements following glutamatergic blockade is mediated by fast changes in RMP

**Figure 8.**
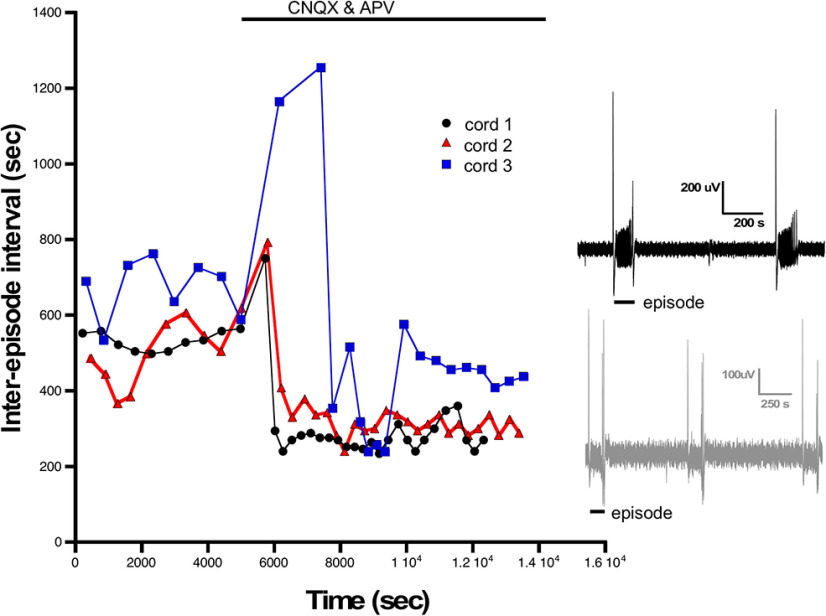
Episodes of SNA are delayed and then recover following glutamatergic blockade. The interval between episodes of SNA are plotted against elapsed time for three different cords before and following treatment with CNQX and APV (20 and 50 μm, respectively). Episode intervals are increased just after adding glutamate receptor antagonists but then recover to a higher rate. Inset shows example traces of SNA from a cord before (black) and after bath addition of CNQX/APV (gray). Traces were filtered from 0.1 Hz to 10 kHz.

**Figure 9.**
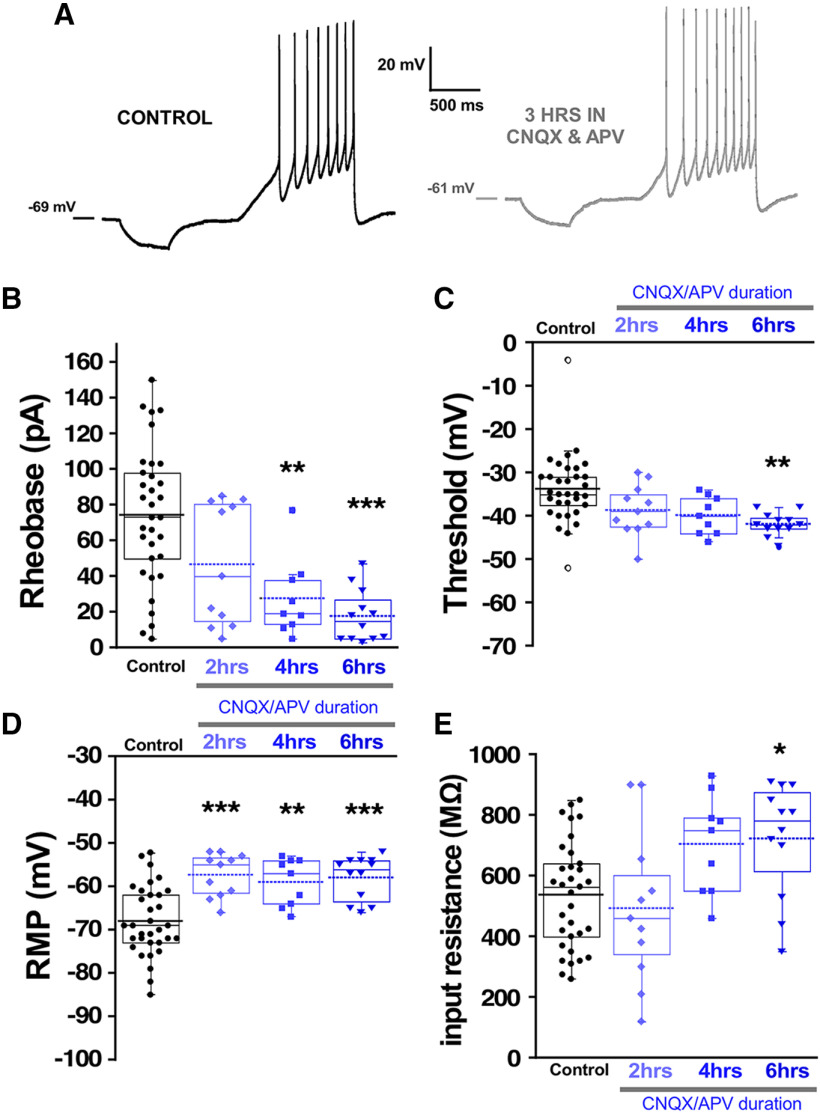
Motoneuron excitability increased following *in vitro* glutamatergic blockade. Motoneuron excitability was measured in isolated spinal cords using a ramp protocol from 0 to 200 pA in 1.2 s to assess rheobase current and voltage threshold in the absence (control, 33 cells; 12 cords) or in the continuous presence of 10 μM CNQX and 50 μM APV at three different blockade periods: 0–2 h (11 cells, four cords), 2–4 h (eight cells, three cords), and 4–6 h (12 cells, four cords). Representative traces of motoneuron firing in control and after 3 h of continuous CNQX and APV in the bath (***A***). Box and dot plots showing quartile distribution and individual values for of the rheobase (***B***), threshold (***C***), RMP (***D***), and input resistance (***E***) in isolated spinal cord motoneurons. For all plots, the continuous line represents the median and the dotted line represent the mean of the sample; *p < 0.05, **p < 0.01, ***p < 0.001, ANOVA with Bonferroni *post hoc* test.

**Figure 10.**
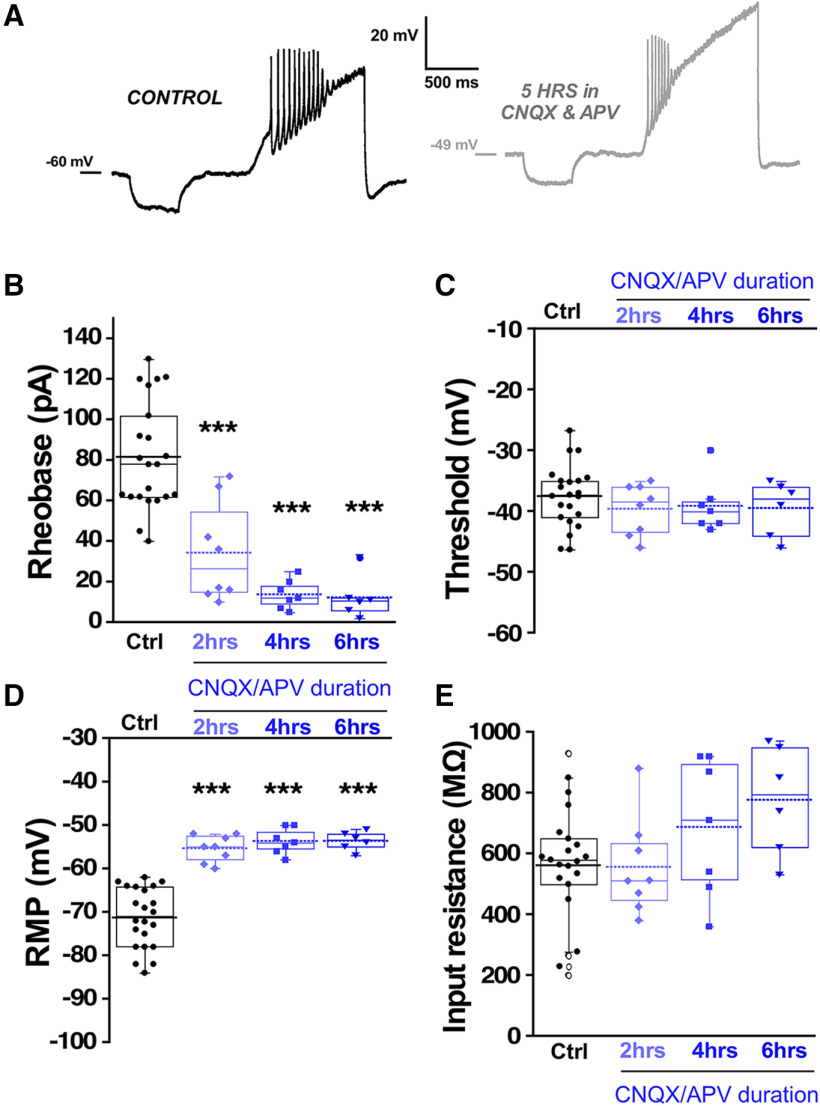
Interneuron excitability increased following *in vitro* glutamatergic blockade. Interneuron excitability was measured in isolated spinal cords with the same protocol used for motoneurons to calculate rheobase current and voltage threshold in controls without drugs (22 cells, eight cords, these are the same values as in Fig. 4 control interneurons) or in the continuous presence of 10 μM CNQX and 50 μM APV at three different blockade periods: 0–2 h (eight cells, three cords), 2–4 h (seven cells, three cords), and 4–6 h (six cells, three cords). ***A***, Representative traces of interneuron firing in control with no drugs and after 5 h of continuous CNQX and APV in the bath. Box and dot plots showing quartile distribution and individual values of rheobase (***B***), threshold (***C***), RMP (***D***), and input resistance (***E***) in isolated spinal cord. For all plots, the continuous line represents the median and the dotted line represent the mean of the sample. Sample size is indicated as (interneurons/chick embryos) in all plots; ***p < 0.001, ANOVA with Bonferroni *post hoc* test.

Previous work has demonstrated that a similar homeostatic recovery of embryonic movements was observed following either glutamatergic or GABAergic blockade (Wilhelm and Wenner, 2008). Movements recovered in around 12 h, but after 12 h of glutamatergic blockade, no synaptic scaling or homeostatic changes in intrinsic excitability were observed. However, it is unknown whether changes in intrinsic excitability occur at earlier time points in the presence of glutamatergic antagonists. Therefore, we isolated spinal cords and applied CNQX (20 μm)/APV (50 μm) to the *in vitro* preparation and monitored the expression of SNA. We saw that glutamatergic blockade delayed the next episode of activity but then recovered to a slightly faster rate than before the drugs were added (Fig. 8). As reported previously (Chub and O'Donovan, 1998), the duration of the episodes of SNA was reduced following bath addition of CNQX/APV (Extended Data Fig. 8-1). We examined the compensatory changes in intrinsic excitability that might mediate the increased excitability of these cords. We assessed intrinsic excitability from 0 to 6 h of glutamate receptor blockade in the continued presence of the antagonists. We observed that motoneurons did indeed express reductions in threshold current from 2 to 6 h and a hyperpolarized threshold voltage at 4–6 h of drug application (Fig. 9*B*,*C*; Table 1). In addition, there was a significant depolarization of RMP (>10 mV) from 0 to 6 h accompanied by no change in input resistance from 0 to 4 h (Fig. 9*D*,*E*; Table 1). Similar changes were observed in interneurons following glutamatergic blockade, however the compensations in threshold voltage and RMP (>15 mV) were even more dramatic, while threshold voltage was slightly hyperpolarized but this did not reach significance (Fig. 10; Table 1). The results suggest fast homeostatic changes in membrane potential significantly contribute to compensatory changes triggered by neurotransmitter receptor blockade. Together, the results focus our attention on homeostatic changes in RMP as contributors to the recovery of embryonic movements during either GABAergic or glutamatergic blockade.

10.1523/ENEURO.0526-19.2020.f8-1 Extended Data Figure 8-1 SNA episode duration is reduced following neurotransmitter receptor blockade. Following the bath addition of CNQX/APV, episode duration is reduced compared to that before adding the drugs. Individual dots represent duration of a single episode before and after drug addition to an individual cord. Download Figure 8-1, TIF file.

## Discussion

Homeostatic mechanisms such as synaptic scaling and changes in voltage-gated conductances are thought to be the main strategies that allow maintenance of activity levels. Here, we describe a new mechanism for homeostatic recovery using compensatory changes in RMP that occurs in the first hours of the perturbation.

### Homeostatic mechanisms and their contribution to the recovery of embryonic activity following neurotransmitter receptor blockade

Synaptic scaling was not expressed at 6 h in motoneurons or interneurons (Fig. 7), and therefore, this form of homeostatic plasticity does not appear to be involved in the recovery of embryonic spinal activity, consistent with previous work (Wilhelm and Wenner, 2008). While scaling does not appear to mediate the recovery in the embryonic spinal cord, it appears to influence recovery of spiking activity in the cortex following *in vivo* sensory deprivation (Hengen et al., 2013; Glazewski et al., 2017; however, see Bridi et al., 2018).

Compensatory changes in homeostatic intrinsic excitability were observed following 6 and 12 h of *in vivo* GABAergic blockade. Significant reductions in threshold current were observed in both motoneurons and interneurons. This appears to be largely due to a hyperpolarization of the threshold voltage. On the other hand, compensatory changes in RMP were not observed, with one exception (interneurons following a 6-h gabazine treatment). Therefore, GABAR blockade *in vivo* triggers clear compensatory changes in threshold voltage at both 6 and 12 h of treatment that can be observed in the isolated cord no longer in the presence of the GABAergic antagonist.

These mechanisms were different when the cord was treated with GABAergic or glutamatergic receptor antagonists *in vitro*, where the drugs remained in place as we made measurements of cellular excitability. We observed some changes in threshold voltage in motoneurons and interneurons, but it was clear that reductions in threshold current were, in this case, largely due to significant depolarizations in RMP (>10 mV), at 0–2, 2–4, and 4–6 h of GABAergic or glutamatergic blockade in both motoneurons and interneurons. The recovery of embryonic movements following GABAergic and glutamatergic blockade were temporally very similar, although the mechanisms appeared to be distinct as compensatory changes in threshold current were not previously observed following a 12-h glutamatergic blockade *in ovo* (Wilhelm et al., 2009). This, therefore, focuses our attention on the fast homeostatic changes in RMP as a critical mechanism of homeostatic recovery of SNA in this developing circuitry.

The spinal interneurons were patched blindly, and so this population will represent a diverse one of many cell classes (Ritter et al., 1999). Therefore, in cases where we did not see a change in the measured parameter, the variability of the populations could contribute to this. Similarly, we did not distinguish among motoneurons projecting to different muscles, and so again this could contribute to variability. Importantly, no matter what kind of interneuron (GABAergic or glutamatergic) or motoneuron (femorotibialis or tibialis anterior) that we recorded from, the change in RMP was a universally observed feature. Further, it is becoming clear that variability is a biological reality as functionally equivalent cells and circuits can achieve their common behaviors using highly different strategies or parameter space solutions (distinct constellations of synaptic and voltage-gated conductances; Marder et al., 2015).

### Homeostatic perturbations and RMP

No changes in RMP were reported in two of the earliest studies on homeostatic plasticity where different strategies were used to chronically block spiking for days (TTX, CNQX, or cell isolation), in rat cortical cultures (Turrigiano et al., 1998) or the stomatogastric neurons of the lobster (Turrigiano et al., 1994). Since these early studies, several other homeostatic experiments have been performed where spiking or neurotransmission were chronically blocked to trigger homeostatic synaptic or intrinsic plasticity and no changes in RMP were observed. These studies have been conducted using various perturbations (TTX, TTX/APV, CNQX, NBQX, gabazine, bicuculline, cell isolation) in rat cortical cultures (Turrigiano et al., 1998), mouse cortical cultures (Bülow et al., 2019), and in the embryonic spinal cord of the chick embryo *in* vivo (Gonzalez-Islas and Wenner, 2006; Wilhelm and Wenner, 2008; Wilhelm et al., 2009). On the other hand, none of these studies followed the RMP before and immediately after the perturbation as we have done in the current study. Therefore, changes in RMP may have occurred in these previous studies but over the duration and/or after removal of the perturbation no change was detected. An exception to this was described following a strong perturbation (two-week exposure to 15 mm KCl), where a homeostatic hyperpolarization of RMP was observed in cultured hippocampal neurons (O'Leary et al., 2010).

### Conductances that contribute to the RMP

Several different ion channels exhibit activation at subthreshold potentials and thus contribute to setting the RMP including multiple kinds of K^+^ channels (Ia, Ikir, Ileak; Coetzee et al., 1999; Plant et al., 2019), hyperpolarization-activated cationic channels (Ih; Robinson and Siegelbaum, 2003; Biel et al., 2009), low-threshold calcium channels (Perez-Reyes, 2003), persistent sodium currents (NaPIC; Crill, 1996; Waxman et al., 2002), and leak sodium channels (NALCN; Lu et al., 2007; Ren, 2011). In addition, ongoing synaptic conductances can also influence the RMP (Mody and Pearce, 2004; Chuang and Reddy, 2018). Previous work has shown that blocking GABARs by direct application of a GABA receptor antagonist onto a chick embryo spinal cord preparation causes an acute hyperpolarization in spinal neurons that can be as large as 10 mV, suggesting a significant tonic GABAergic depolarizing current (Chub and O'Donovan, 2001). The effect of acute application of GABAergic antagonist onto the cord (Chub and O'Donovan, 2001) is in the opposite direction (hyperpolarizing) compared with the current studies finding that bath application of gabazine leads to a depolarization of RMP in the first hours of drug exposure. The current study is the only one we are aware of that follows RMP before and throughout the first hours of the perturbation and may explain why this form of homeostatic intrinsic plasticity has not been previously reported.

### Mechanisms of early homeostatic changes following neurotransmitter receptor blockade

What is a potential trigger for these homeostatic changes in RMP? It has been shown previously that homeostatic synaptic scaling is triggered following 48 h block of GABAergic transmission (Wilhelm and Wenner, 2008) and compensatory changes in voltage-gated ion channel conductances by 12 h of GABAR block (Wilhelm et al., 2009). In fact, merely altering GABAR activation due to spontaneous release of GABA vesicles can fully trigger synaptic scaling (Garcia-Bereguiain et al., 2016). However, compensatory changes in RMP were not so reliant on GABAR activation. Fast changes in RMP were not triggered by altering the frequency of spontaneous vesicle-mediated GABAR activation (Fig. 6). Further, these changes can also be triggered by reduced glutamatergic receptor activation where GABAR activation is intact. Therefore, the most straightforward explanation for the trigger of homeostatic changes in RMP would be the reduction in network activity caused by blocking either glutamatergic or GABAergic receptors.

A commonly described mechanism underlying a change in RMP involves a change in some resting channel conductance (e.g., K^+^ channels), however we did not detect a change in input resistance, making this possibility less likely. A potentially more plausible mechanism would involve a change in the function of the Na^+^/K^+^ ATPase. Previous studies are consistent with this possibility. First, RMP of invertebrate neurons can be changed by an alteration of the electrogenic Na^+^/K^+^ ATPase (Carpenter and Alving, 1968; Carpenter, 1973; Willis et al., 1974). Next, work in the spinal cord of *Xenopus* and neonatal mice, as well as in motoneurons of the fly larva, show that bursts of spiking activity expressed in these systems lead to an increase in intracellular Na^+^, that is necessary to activate an isoform of the Na^+^/K^+^ ATPase that is not active at baseline Na^+^ levels. The Na^+^-dependent activation of this Na^+^/K^+^ ATPase produces a hyperpolarizing current due to the electrogenic nature of the pump that has been called an ultra-slow afterhyperpolarization (usAHP; Pulver and Griffith, 2010; Zhang and Sillar, 2012; Picton et al., 2017a,b). This hyperpolarizing current is maintained for up to a minute. SNA in the chick embryo spinal preparation experiences a very similar usAHP after episodes of SNA (O'Donovan, 1999; Chub and O'Donovan, 2001). Further, embryonic spinal neurons have very high Na^+^ concentrations at baseline (Lindsly et al., 2017). Therefore, it is possible that Na^+^ levels constitutively activate this Na^+^/K^+^ ATPase and when SNA is blocked for many minutes by glutamatergic or GABAergic antagonists, Na^+^ levels eventually are reduced to a point that pump activity is minimized and the hyperpolarizing current abates, thus depolarizing the RMP.

Most of our results appear to suggest that the changes in RMP are expressed transiently while the antagonists are in place, and once washed off the RMP returns to pre-drug levels. This kind of temporary perturbation might not permanently change the developmental trajectory of spinal neurons or their network. However, if this initial fast homeostatic mechanism does not recover activity levels or is maintained for long periods, then other mechanisms may be triggered, which could alter the development of the spinal circuitry in a long-lasting manner. This may be the case following GABAergic blockade where compensatory changes in threshold voltage are triggered *in vivo* and *in vitro*. This is consistent with previous 12-h GABAergic blockade *in vivo* where threshold current is dramatically reduced by compensatory changes in voltage-gated Na^+^ and K^+^ channels (Wilhelm et al., 2009). Changes in protein levels of two of these previously implicated voltage-gated ion channels were observed at 12 h, although not at 6 h of *in vivo* gabazine treatment (Fig. 5). In some cases, homeostatic changes in RMP may be sufficient and exist temporarily, but in other cases these additional mechanisms could be engaged to recover the activity.

## Synthesis

Reviewing Editor: Juan Burrone, King’s College London Decisions are customarily a result of the Reviewing Editor and the peer reviewers coming together and discussing their recommendations until a consensus is reached. When revisions are invited, a fact-based synthesis statement explaining their decision and outlining what is needed to prepare a revision will be listed below. The following reviewer(s) agreed to reveal their identity: Niraj Desai.

The authors have done a very good job of answering all queries raised by the reviewers. Some minor comments are included below for the authors to consider but the paper is deemed ready for publication as is.

Reviewer 1

I would like to extend my congratulations to the authors for massively improving the manuscript. I particularly appreciate the care that has been put in providing extensive methodological details, and in reworking the figures.

I have three remaining very minor comments, which are really only suggestions and not requests. I am happy to leave to authors and editor a final decision on these minutiae.

1) In my personal opinion, I do not think that you can call synaptic events “miniature” if they were recorded in absence of TTX. I would suggest to change nomenclature and identify them as “spontaneous”, sPSCs instead of mPSCs. However, if this is not done in the spinal cord world, now that it is clearly stated in the methods that TTX was not applied, I can live with mPSCs.

2) Current clamp step protocols. Thank you for including details on step and ramp protocols. One thing is still unclear to me: were criteria for holding voltage applied to accept or reject each current step? e.g., only steps that started at holding Vm plus/minus XX mV were accepted. It may be not needed in the cord, but many cells, especially autorhythmic interneurons tend to slip the clamp during the protocol, and clearly a different baseline Vm results in a biased current step. Whatever you did, a few words to explain it would suffice

3) Synaptic events charge relates not just to amplitude but also to kinetics. You could have a change in charge with same amplitude if decay time changes (see fig 7 GABA events example trace). You may have an undetected phenotype, but if you do not want to check/agree with the measure, ignore this.

Reviewer 2

I’m satisfied with these revisions. The variability still troubles me, but it is what it is; I don’t have other suggestions on that score.

I’m glad the authors added scatter plots. I wouldn’t insist on estimation statistics in this case. The scatter of the data already gives a sense of what estimation statistics would indicate.

## Author Response

We thank the reviewers/reviewing editor for their diligent review of our manuscript. We have addressed the reviewers’ comments and believe this has improved the paper significantly. Below, in blue, we answer the concerns of the reviewers/editor. We also provide a copy of the manuscript with changes in blue and a clean copy. Thanks again.

Synthesis Statement for Author (Required):

This study addresses an important question in the field of homeostatic plasticity. It uses spinal cord neurons to assess the mechanisms that allow the recovery of spontaneous network activity following blockade of synaptic transmission. The findings are potentially interesting and uncover changes in the resting membrane potential as a novel target for compensatory mechanisms, together with changes in firing threshold, both of which are proposed to modulate overall neuronal excitability. Although certainly interesting, a number of issues have been raised by the reviewers that the authors should address. These include providing more methodological detail, providing clear statistical methods (here I would recommend the use of estimation statistics) and displaying data to visualise the distribution of all data points, as well as include example traces. The latter point is particularly important to better understand the heterogeneity observed in the data, especially across time and neuron type. In addition, although the authors attempt to establish a mechanism for the observed changes in intrinsic properties, more could be done here to understand the channels that play a role in this form of plasticity, which would also help better interpret their findings. Overall, this is potentially a very interesting story. Below I have included the specific comments of the reviewers for the authors to address. Note that there is some overlap in the comments by reviewers and the authors should feel free to answer by topic to avoid repetition.

We have added considerable methodological detail, we provide clear statistical analysis and show the data as scatter plots superimposed on box plots to better display the actual data, and we have included example traces in all the figures. As discussed with the editor we are not going further in defining mechanism as this is not a requirement for eNeuro, but mechanism will be fully addressed in a future paper.

Reviewer 1

"The question is of great interest” - Thank you.

- The main finding of this paper is that pharmacological blockade of synaptic transmission is counteracted by changes in the cell intrinsic excitability via changes to the resting membrane potential (Vm). I have scanned the paper for it, but I was unable to find a detailed description of exactly how they measured Vm. Was is done immediately after break in current clamp, or in voltage clamp via zero-current potential, or in some other way? Was liquid junction potential corrected for? Having an accurate reading of Vm in whole-cell mode is not a trivial thing, and since their main finding hinges on it, the authors should really make a convincing case for their chosen method.

RMP was measured in current clamp, ∼5 min after breakthrough in order to let the recording stabilize in voltage clamp mode first. We now describe this in the manuscript. Further, we thank the reviewer for the suggestion to correct for the liquid junction potential, which was surprisingly large (12mV) and show the results as a scatter plot so the readers can appreciate the clear change that is observed. The other thing we have now added are the results from control cords that were never treated with an antagonist but recorded from 0-2, 2-4, and 4-6hrs after starting the recordings. We show that these values do not change significantly over this period showing that the depolarization in RMP is not merely a time dependent process.

- Overall, much more detail is needed for all methodology. A few examples:

o Were experimenters blinded to treatment? Was the analysis, or parts of it, done blindly?

The experiments were not blinded. However, the main finding of the change in RMP was not initially appreciated as we were not focusing on this physiologic phenotype, but rather on the rheobase and other intrinsic excitability measurements. So, in this sense we did not have any bias as we did not expect to see changes in RMP. We now include a statement that the study was not blinded.

o How were the western blot quantified? How many chicks were processed?

Films were scanned and analyzed using Image J with background correction and normalization to actin. Each sample is from 4 cords/chicks per treatment and this was done in duplicate. This is now stated more clearly in the manuscript.

o How were the minies analysed? Inclusion/exclusion criteria based on noise value?

Fully automated choice done by mini analysis, and if so, with which settings?

Or was minis analysis supplemented by some input from experimenter, and if so, was a small set of experiments double-checked by second person?

We have now added a detailed description of mini analysis to the Methods section (Electrophysiology) addressing all of these concerns. There was no second person analyzing the data, however we have done this in the past and have not observed significant differences, even when using Axograph instead of Minianalysis (Synpatosoft software).

o Clearly state for both motor neurons AND interneurons all inclusion criteria used (Rs, Rm, Ihold - relative and absolute).

We had put in the following text initially but now highlight in blue - “Recordings in current clamp were terminated whenever significant increases in input resistance (>20%) occurred. For voltage clamp experiments, recordings were terminated whenever significant increases in series resistance (> 20%) occurred.” We did not record cells with a RMP more depolarized than -52mV, Ihold of more than 50 pA, and we have added this to the methods.

o How were the data acquired? Filtering, sampling, capacitance compensation etc.

This has been added to the Methods section - Electrophysiology.

o More details on the current clamp stimulation protocols. For instance, in the methods the current steps are indicated as 1s long, but the only trace provided in fig.1 show steps of 500ms. Was a criteria for holding voltage applied to accept or reject each current step? What was the inter-injection interval?

These details have been added to the Methods section - Electrophysiology.

- Statistics and overall reporting of values. Averages, measure of variance and p-values (with relative tests) need to be reported in the results section. Number of animals (N) AND number of cells (n) need to be included in all figure legends. Also, if N is much smaller than n, I would advise on adopting nested statistics (see Aarts E et al, 2014, Nat Neuro).

These values have been added to the Figure legends and Methods section - Electrophysiology.

- Figures. All figures must include both example traces and summary data. This is sometimes done (Fig.1 and 7), but often there are only summary data (Fig. 2, 4, 6, 9, 10). Figure 5 only has one example blot, but the result section reports quantifications. I find figs 3 and 8 difficult to interpret: once more, example traces would help, and maybe a different way of plotting the three cords, either with connecting lines or on three separate graphs? Finally, I am not a fan of bar plots, especially when n is low: I would recommend to use scatter plots (and I believe that this is what JNeuroscience also asks of authors).

We now have example traces for all figures and we have plotted the graphs as a scatter plot on top of a box plot to more thoroughly display the data. This now more clearly shows the data and makes a particularly strong case for changes in RMP. We have better described how the blots were carried out and have added lines to figs 3 and 8.

- SNA bursts: besides the inter-burst interval, was the length of bursts analysed?

It was previously reported that following glutamatergic or GABAergic blockers the duration was reduced, presumably because a significant part of the excitatory synaptic drive was removed. We now cite this publication (Chub et al., 1998) and have added extended data (Fig 3-1, Fig 8-1) showing this in the current study.

- DHBE: it would be very much appreciated to be able to see a trace showing that the drug actually worked, and some quantification of by how much it decreased release.

This was shown in our previous paper (Gonzalez-Islas et al. 2016 Dev. Neurobiol. PMID:26061781). We now describe this in more detail in the current manuscript. We did not assess DHBE’s effect on mini frequency in the recordings for the current study as we measured intrinsic excitability/RMP in current clamp.

- Minies. Was TTX present in the bath? No, and this is now described and justified in the methods.

Besides the reference, could you please provide details on how AMPA mPSCs and GABA mPSCs were dissociated? We now provide a more detailed description of this in the methods (Electrophysiology).

Also, since you clearly analysed kinetics, could you plot them/give values? This has now been included in extended data (Fig 7-1).

Do they correlate with the different potassium channels expression? Not clear what the reviewer is asking here, as we have no measure of K channel current or protein in these experiments. Regardless, the story here was that mPSC amplitudes were not altered at 6 or 12 hours of gabazine treatment and so did not contribute to recovery.

Also, amplitude is not always the most representative measure for postsynaptic function - indeed, the traces presented in 7A for GABA mPSCs look different to me in terms of charge. We now show no change in mini decay (or amplitude) which argues against a change in charge. The example mPSC traces are averages from one cell and vary across cells considerably (this is now mentioned in the Figure 7 legend).

- As a non-specialist on chick spinal cord circuitry, I would like a little more details on the interneurons that the authors recorded from. Is there only a class of interneurons in there, all of the same size (see Cm impact on Ithreshold) and with the same channel composition and properties? If so, please clearly state it and give some references. If not, how did you choose which interneurons to patch, and how did to you control for variability?

The spinal interneurons were patched blindly, so we do not know the specific class of cell we are recording from (see Ritter et. al. J. Neurosci. PMID:10212306 for an analysis of these different classes of interneurons). Therefore, in cases where we do not see a change in the measured parameter, the variability of the populations could contribute to this, and the same case could be made for motoneurons projecting to different muscles. The important observation here is that no matter what kind of interneuron (GABAergic or glutamatergic) or motoneuron (femorotibialis or tibialis anterior) the change in resting membrane potential was a universal one. We have elaborated on this now in the text of the manuscript.

- Check the text for missing references. For instance, I would like some in line 296.

We have added this reference as well as others throughout the text.

Reviewer 2

"The scientific questions under study here are important” - “and the finding on resting membrane potential very interesting” -

Thank you

DATA VARIABILITY: The approach employed here is quite thorough, but the manuscript often comes off like a hodgepodge of results that are hard to make sense of. For example, when measured in vitro (Fig. 3), both motoneurons and interneurons have depolarized resting potentials after a short (0-2 h) GABAzine treatment, but when measured in vivo (treated in vivo, there were no measurements in vivo of RMP) (Fig. 2), only interneurons show this effect and then only at a single time point (6 h but not 12 h). What are readers supposed to make of this?

We did not make this point clearly enough. The in vitro recordings are done in the presence of gabazine and we believe this is the critical factor. The recordings following in ovo gabazine treatment (6 and 12hrs) were still measured in vitro but were not done in the presence of the gabazine. The difference of 6 and 12 hrs in vivo could be due to variability as described below.

To cite a different example, in vitro motoneurons show a drop in threshold potential at the 2-4 h time point, but not the 0-2 h point or the 4-6 h point. Again, should the reader take this seriously?

Real experimental data are messy, I realize, and the in vivo preparation has some fundamental differences from the in vitro preparation, as the authors point out, but the randomness of some of the results is worrisome. This is especially true because one of the design goals of this study was to track the time course of compensatory changes in response to activity perturbation. If random effects show up, it’s hard to have confidence in the time course.

We agree that there is variability. As mentioned by the first reviewer, we are dealing with a variable population of interneurons with different functions and neurotransmitters and even our motoneurons project to different muscles, so this has likely contributed to our variability. However, our main message is the change in the RMP, which was quite consistent across all types of neurons giving us confidence in the robustness of this finding. We have slightly de-emphasized the changes in threshold voltage and added a discussion about variability. Further, Eve Marder and colleagues have demonstrated that variability is a biological reality as functionally equivalent cells and circuits achieve homeostasis using quite different strategies and we have now added this consideration to the discussion.

One (relatively) simple thing the authors could do to address this point is to show all the data. At present, the manuscript relies on bar graphs with standard errors to represent the data. But the trend in contemporary neuroscience papers, especially at eNeuro, is to show individual data points (e.g., in a scatter plot) and not just averaged data. In fact, this manuscript seems like an ideal place for “estimation statistics” to be employed, which would provide a visual representation of the confidence intervals and provide a much better idea to the reader of what reported differences mean. Using estimation statistics seems to me a very good starting point for trying to sort through data variability.

We have now changed the bar charts to scatter plots on top of box and whisker plots to better display the data, and which makes a very strong case for the changes in RMP. We have applied standard statistical tests which are used routinely in these kinds of studies.

CORRELATIONS: The manuscript reports standard measurements of intrinsic excitability: resting potential, threshold (rheobase) current, threshold potential, resting input resistance. But these measurements are not independent of each other, as the authors realize as they point this out at a couple of places. Most simply, one would expect threshold current to covary with resting potential and especially input resistance, but one might also expect threshold potential to covary *somehow* with resting potential, if, for example, a different mix of channels were active at rest. It would have been better to measure these quantities both at a neuron’s own resting potential and at a common membrane potential (maintained by DC current) to disentangle these correlations. (A side point: the authors use current steps for in vivo measurements and current ramps for in vitro measurements. Why?)

We looked at all correlations and surprisingly nothing clear came out of this. As the reviewer suggested there were cases where a correlation was observed such as RMP and rheobase (3 out of 16 cases - 4 conditions (con, 0-2, 2-4, 4-6hrs) X 2 cell types (MNs or INs) X 2 treatments (GluR or GABAR block)). However, these were relatively rare and not in any obvious pattern and were not observed in control conditions, were we had the largest pool of observations. We have added the table below showing this but were not planning on adding it to the manuscript since there were no general trends. We could put this data as extended data if the reviewers think this is worthwhile. Again, this may have something to do with the natural variability that is observed across cells. As for the different current injection protocols, the in vivo current measurements took considerably longer than the ramps that were done in vitro. With the in vitro measurements we wanted to record from cells within a specific time window, so it was important to do this as quickly as possible. With the in vivo recordings we obtained ∼3 cells per cord over 3-4 hours, so there was it was not difficult to use the preferred but slower step protocol. We now mention this in the methods.

This is another issue that might be addressed if readers could see individual data points. For example, plot resting input resistance on the x-axis and threshold current (or potential) on the y-axis. There should exist a correlation and it would be useful for the reader to see how the correlation differs between treatment groups. The manuscript reports that resting input resistance was not affected in any treatment group, but there is clearly a lot of variation in input resistance values between neurons of a single group, given the standard errors (it’s hard to look at Fig. 4D and think “the differences between those bar plots, apparent to my eye, mean nothing.”)

We now plot all data as a scatter plot of data on top of a box and whisker plot.

MECHANISMS: The single thing that would strengthen this manuscript most would be an investigation of the mechanism(s) behind the resting membrane potential result. The Western blots of Fig. 5 address this issue to some extent, but much more could - and, in my opinion, should - be done. Fig. 5 shows that GABAzine increases Nav 1.2 after 12 h of treatment, but not 6 h. So, what accounts for the threshold potential changes displayed in Fig. 4? Likewise, Kv 4.2 is decreased at 12 h but not 6 h. So, it also doesn’t explain earlier results. In the Discussion, the Na/K pump is offered as an explanation, but no actual data in this paper provides evidence for this explanation. Nor are the currents carried by Nav 1.2 or Kv 4.2 actually measured. Consequently, readers are left with an array of results that are interesting, but hard to judge or interpret.

The physiologically identified channel currents that mediate changes following 12 hour in ovo gabazine treatment have been described by us previously (Wilhelm et al., 2009, PNAS PMID:19346492). This is now better highlighted in the manuscript. We do not know what channels mediate changes that occur following {less than or equal to}6hr antagonist treatment in the in vitro studies, but this is not the focus of the current manuscript, and as mentioned we have somewhat de-emphasized this part of the study. Rather, we are focusing on the homeostatic changes in RMP. In the criteria section of the overview of eNeuro it is stated that mechanistic insight is not needed if the finding represents an important observation that moves the field forward (in our case homeostatic plasticity). The reviewers seemed to suggest the finding is important (reviews 1 - “The question is of great interest", reviewer 2 - “The scientific questions under study here are important - and the finding on resting membrane potential very interesting”). We do show a mechanism which is a shift in the resting membrane potential, but do not show a molecular mechanism, but from our reading of the criteria of eNeuro it seems as though we have achieved what is necessary - an important observation. Showing that the Na-K pump is involved in a substantial way would require a significant amount of additional studies which we plan to make in a completely separate study, rather than simply adding the first piece of this argument to the current paper.

eNeuro - Overview: Criteria

"To warrant publication in eNeuro, a manuscript must provide information that is important to the targeted scientific audience. Importance does not necessarily mean mechanistic insight. It can be an observation, but an important one that can move a field forward.”

OTHER: (1) What statistics were used to test the f-I curves of Fig. 1? Repeated measures ANOVA or something else? (2) In Fig. 4B, why not probe a 2-4 h time point?

(1) We had not done statistics on the FI curve. We now have collapsed steps from 90-110pAs into a data point and compare with a one way ANOVA across the 3 conditions. (2) There is a 2-4 hour time point, and it was not significantly different than control, but we are not 100% sure what the reviewer is referring to in terms of probing this.
