# Supplementary figures and images for: Homeostatic Recovery of Embryonic Spinal Activity Initiated by Compensatory Changes in Resting Membrane Potential
Source: eNeuro. 2020 Jul 7;7(4):ENEURO.0526-19.2020. doi: 10.1523/ENEURO.0526-19.2020 (PMC7340840; doi:10.1523/ENEURO.0526-19.2020)

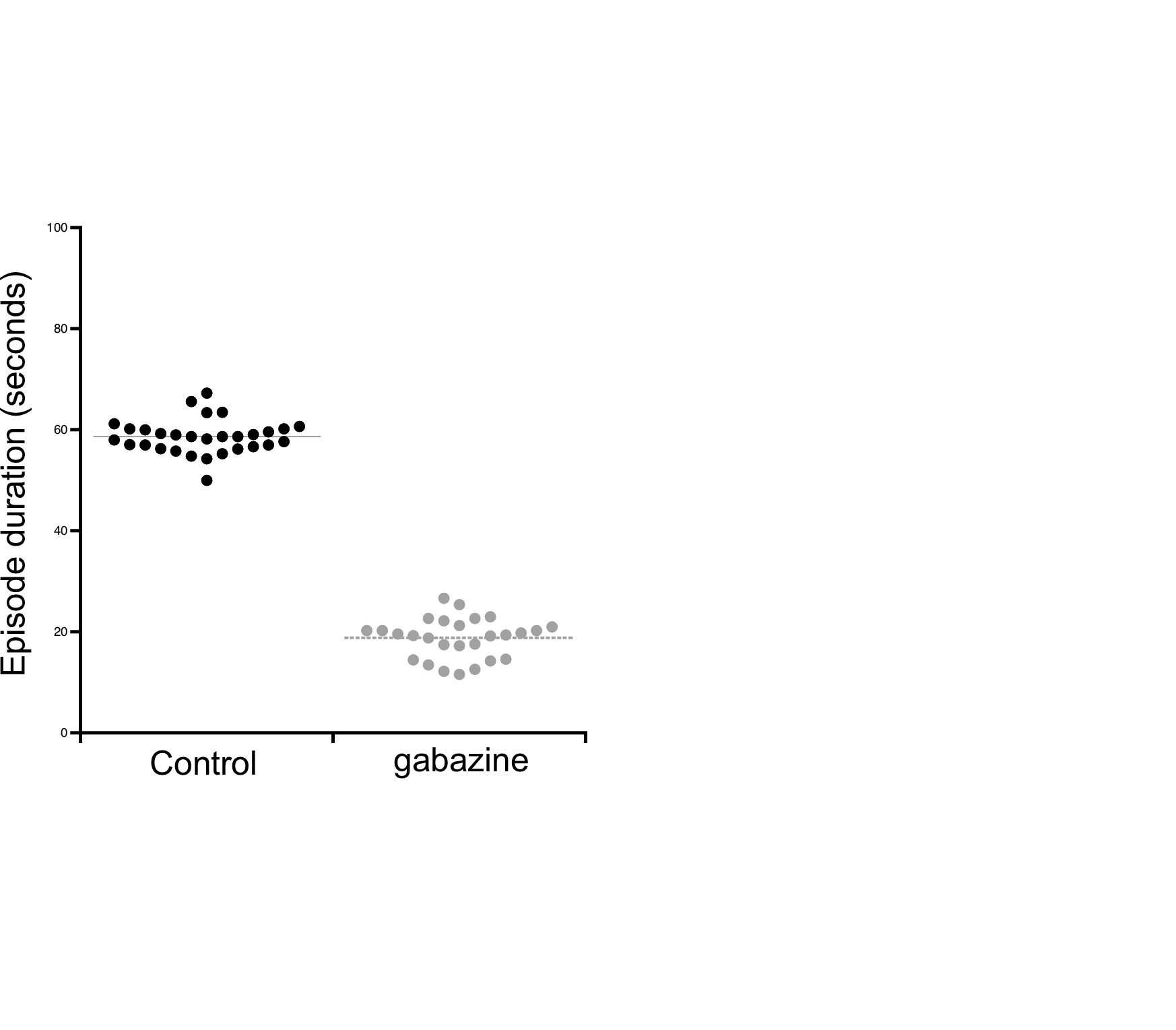

Supplement: Extended Data Figure 3-1 — SNA episode duration is reduced following neurotransmitter receptor blockade. Following the bath addition of gabazine, episode duration is reduced compared to that before adding the drug. Individual dots represent duration of a single episode before and after drug addition to an individual cord. Download Figure 3-1, TIF file. [file enu-eN-NWR-0526-19-s02.tif]

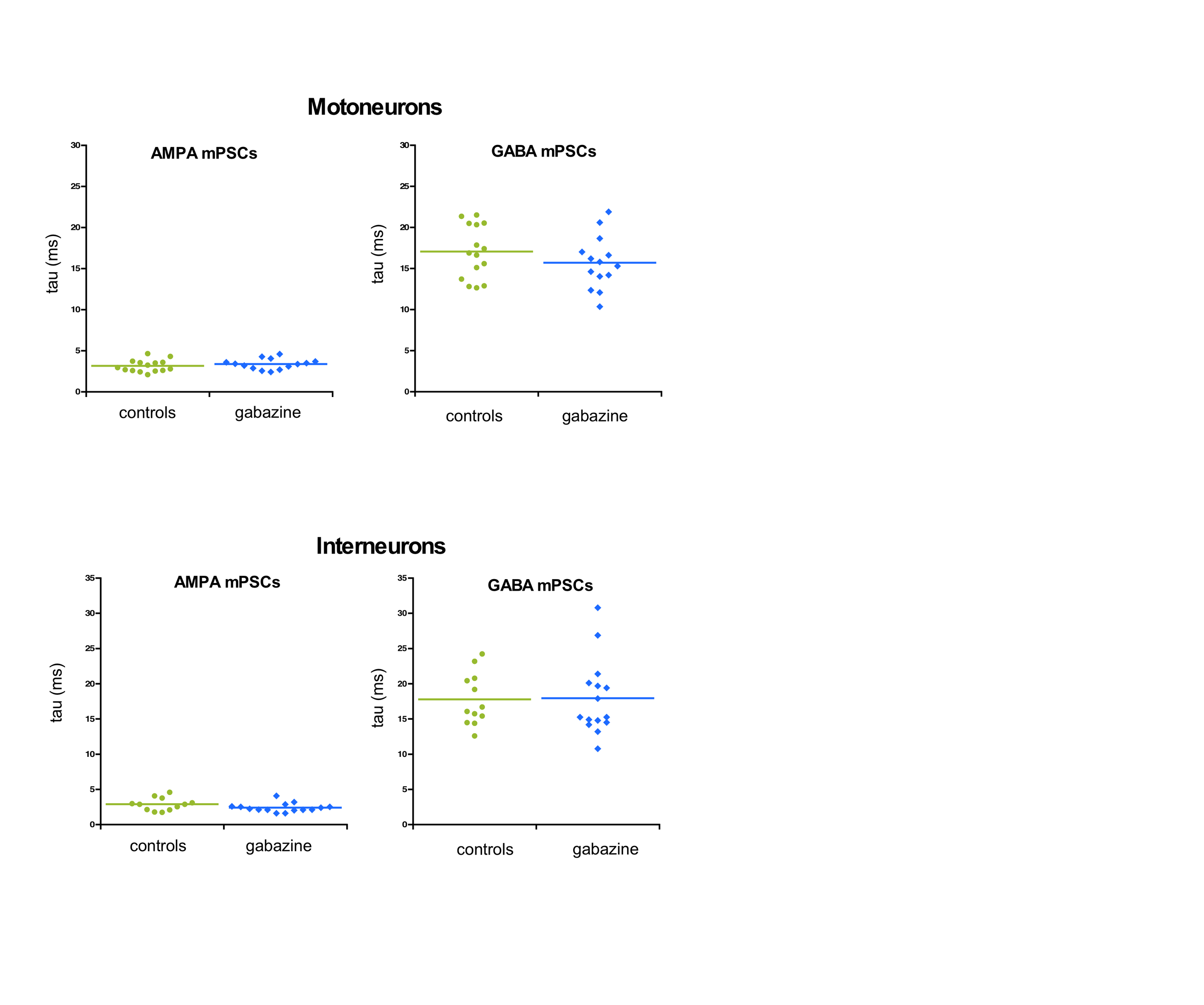

Supplement: Extended Data Figure 7-1 — Decay time constants (τ) for GABA mPSCs (A) before (τ =17.06 ± 0.83 ms; n = 15) and (B) after (τ = 15.7 ± 0.86 ms; n = 14) the addition of gabazine to the bath were not significantly different (p = 0.26). τ for AMPA mPSCs (C) before (τ = 3.16 ± 0.19 ms; n = 15) and (D) after addition of gabazine to the bath (τ = 3.39 ± 0.17 ms; n = 14) are shown. No significant difference was observed (p = 0.39). Download Figure 7-1, TIF file. [file enu-eN-NWR-0526-19-s03.tif]

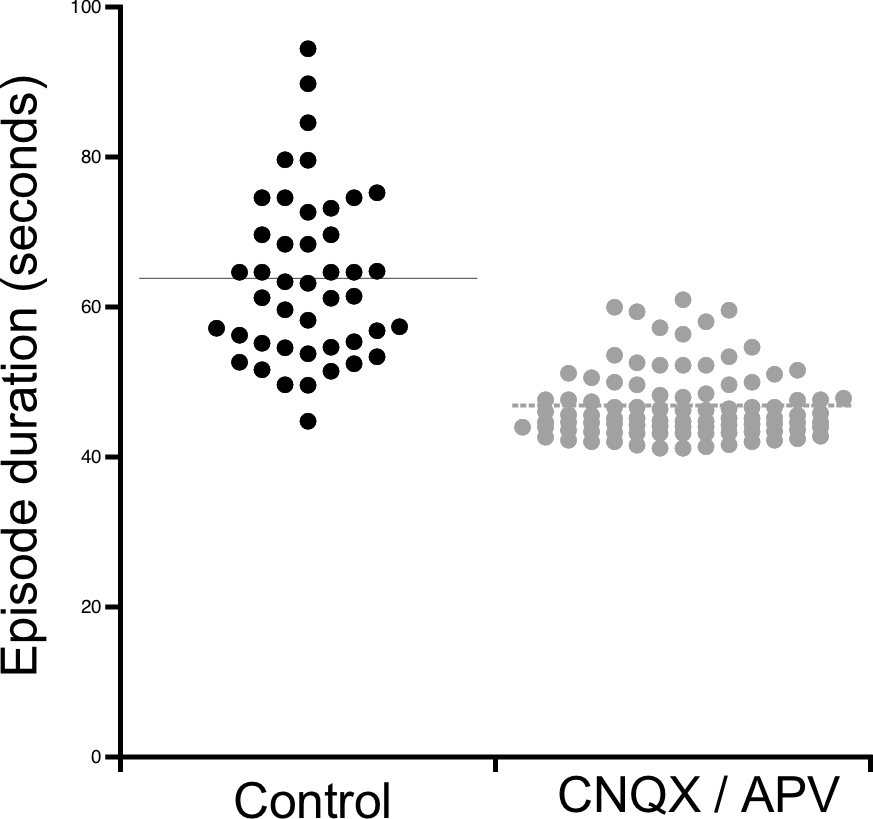

Supplement: Extended Data Figure 8-1 — SNA episode duration is reduced following neurotransmitter receptor blockade. Following the bath addition of CNQX/APV, episode duration is reduced compared to that before adding the drugs. Individual dots represent duration of a single episode before and after drug addition to an individual cord. Download Figure 8-1, TIF file. [file enu-eN-NWR-0526-19-s04.tif]
